# Spatial Heterogeneity in Drug Concentrations Can Facilitate the Emergence of Resistance to Cancer Therapy

**DOI:** 10.1371/journal.pcbi.1004142

**Published:** 2015-03-19

**Authors:** Feng Fu, Martin A. Nowak, Sebastian Bonhoeffer

**Affiliations:** 1 Theoretical Biology Group, Institute of Integrative Biology, ETH Zurich, Zurich, Switzerland; 2 Program for Evolutionary Dynamics, Department of Organismic and Evolutionary Biology, Department of Mathematics, Harvard University, Cambridge, Massachusetts, United States of America; Emory University, UNITED STATES

## Abstract

Acquired resistance is one of the major barriers to successful cancer therapy. The development of resistance is commonly attributed to genetic heterogeneity. However, heterogeneity of drug penetration of the tumor microenvironment both on the microscopic level within solid tumors as well as on the macroscopic level across metastases may also contribute to acquired drug resistance. Here we use mathematical models to investigate the effect of drug heterogeneity on the probability of escape from treatment and the time to resistance. Specifically we address scenarios with sufficiently potent therapies that suppress growth of all preexisting genetic variants in the compartment with the highest possible drug concentration. To study the joint effect of drug heterogeneity, growth rate, and evolution of resistance, we analyze a multi-type stochastic branching process describing growth of cancer cells in multiple compartments with different drug concentrations and limited migration between compartments. We show that resistance is likely to arise first in the sanctuary compartment with poor drug penetrations and from there populate non-sanctuary compartments with high drug concentrations. Moreover, we show that only below a threshold rate of cell migration does spatial heterogeneity accelerate resistance evolution, otherwise deterring drug resistance with excessively high migration rates. Our results provide new insights into understanding why cancers tend to quickly become resistant, and that cell migration and the presence of sanctuary sites with little drug exposure are essential to this end.

## Author Summary

Failure of cancer therapy is commonly attributed to the outgrowth of pre-existing resistant mutants already present *prior to* treatment, yet there is increasing evidence that the tumor microenvironment influences cell sensitivity to drugs and thus mediates the evolution of resistance *during* treatment. Here, we take into consideration important aspects of the tumor microenvironment, including spatial drug gradients and differential rates of cell proliferation. We show that the dependence of fitness on space together with cell migration facilitates the emergence of acquired resistance. Our analysis indicates that resistant cells that are selected for in compartments with high concentrations are likely to disseminate from sanctuary sites where they first acquire resistance preceding migration. The results suggest that it would be helpful to improve clinical outcomes by combining targeted therapy with anti-metastatic treatment aimed at constraining cell motility as well as by enhancing drug transportation and distribution throughout all metastatic compartments.

## Introduction

Cancer is a common genetic disease that results from accumulated (epi-)genetic changes in tumor cells [1–5]. Targeted cancer therapy is currently an area of active research [6], and is under rapid development [7–10]. Targeted agents can send cancer into remission, but the response is often short-lived [7–11]. The recurrence of cancer, if treated with single agents, is almost certain due to acquired drug resistance [12, 13]. Among efforts to understand the rapid acquisition of resistance by cancer cells, particular attention has been paid to the pre-existing resistance arising *prior to* treatment [14–17].

In parallel, there has been growing interest in studying how the tumor microenvironment influences cell sensitivity to drugs and thus mediates the evolution of resistance *during* treatment [18–23]. Important aspects of the tumor microenvironment include spatial drug gradients and differential rates of cell proliferation [22, 23]. The heterogeneity of this kind can be found both on the microscopic level within solid tumors as well as on the macroscopic level across metastases [20, 22, 23]. It is not uncommon that incomplete drug distributions within and across metastatic lesions can compromise the efficacy of treatment [22, 23], which may be in part due to the differences in the ability of the drug to penetrate different tissues [24]. As shown in recent experimental and theoretical studies [25–28], drug gradients can help accelerate the evolution of antibiotic resistance. Moreover, a most recent overview of clinical and pharmacological data concerning distribution of many anticancer drugs in human solid tumors highlights the likely importance of insufficient and/or heterogeneous exposure of cancer cells to effective drug levels in tumor resistance [29]. Thus, in order to improve efficacy of cancer therapy, it is highly relevant to investigate how heterogeneous levels of drug distribution in different parts of the tumor or across metastases affect the emergence of resistance.

Amounting evidence suggests metastasis, at least for some cancers, is an early event during primary tumor development [18, 30–35]. By activating tissue invasion and metastases [36], cancer cells are able to escape from the primary site and disseminate to distant parts of the body, causing life-threatening health problems [37, 38]. At the time of diagnosis and treatment (which generally occur late in the course of disease), a high proportion of common cancer patients have already had tumor cells disseminated to distant sites for years prior to presentation [30, 39]. What is more, clinical outcomes are often complicated by the presence of overt or occult micrometastases in patients [30, 39–42]. It is, therefore, of primary interest to understand the emergence of resistance, particularly in the setting of disseminated cancer.

A recent study reported that circulating tumor cells are detected in 13 out of 36 breast cancer survivors 7–22 years after receiving mastectomy [43]. This observation suggests that disseminated cancer, rather than only the primary tumor *in situ*, is actually under stress when treatment is started [30, 39, 41]. Moreover, a few studies observed that metastatic cells tend to quickly become chemoresistant [21, 44–49], suggesting a positive relationship between metastatic phenotype and the rapid acquisition of drug resistance. It is possible that, with the migration and seeding dynamics [31, 34, 39, 50–53], these metastatic cells located at distant metastatic compartments can re-seed each other, particularly in the presence of tumor sanctuary sites with very little drug exposure. Therefore, it is necessary to explicitly account for the role that the metapopulation structure of metastatic disease plays in the rapid emergence of resistance.

Most recently, in an *in vitro* experiment with metastatic breast cancer cells [54], it was shown that cell motility and drug gradient of chemotherapy together can lead to fast emerging resistant cells in areas of high concentrations that would otherwise completely inhibit cell growth. This important experimental result begs theoretical questions aimed at revealing the relevant pathways of evolving resistance. Do tumor cells first migrate from low concentration areas and then adapt to high concentration areas? Or alternatively, do they first acquire resistance in low concentration areas and then migrate to and populate areas of exceedingly high concentrations? How do cell motility and drug gradient, or more generally how does the spatial heterogeneity in drug concentrations affect the emergence of resistance? The goal of the present work is to answer these questions and provide qualitative insights by a simple conceptual model.

Here we focus on the macroscopic level of the tumor microenvironment across metastases, explicitly taking into account the roles of the multi-compartment structure of metastases and cell migration in the emergence of acquired resistance during treatment. Spatial compartments mean different locations that harbor metastatic deposits (i.e., target organs, such as bone marrow, liver, brain and lung) [34]; migration means dissemination and seeding of cells between metastatic compartments [35]. We focus on the common population dynamics that govern the evolution of resistance for various cancers differing in their capacity to metastasize. Metastases of solid tumors (such as breast cancer [42] and melanoma [10]) tend to have well-defined spatial compartments because of low dissemination rate, whereas the compartment structure of liquid cancer (e.g. blood tumors [7]) is diminished by exceedingly high fluidity. By adjusting the migration rate, our model can be suited to study the specific kind of cancer in question.

Clinical observations from multiple sources affirm that an exponential growth model, although remaining an issue of debate [55], is able to adequately describe tumor growth for most cancer patients [56–62]. In line with this, we use a stochastic, multi-type branching model to account for the fate of individual cells, particularly these drug-resistant mutations in establishing surviving lineages. Mathematical models of this kind have provided particularly useful insights into understanding evolutionary dynamics of cancer in response to treatment [11, 14–17, 63–66] (see a review in Ref. [67]). A large set of previous models are focused on pre-existing resistance in the primary tumor, arising from neutral evolution prior to treatment [15, 17]. Built on these prior studies, the current work incorporates the compartment structure of metastatic disease and quantifies the role spatial heterogeneity in drug concentrations plays in the evolution of resistance by metastatic cells during treatment.

In our model, cancer cells can migrate from one spatial compartment to another. Spatial heterogeneity in drug concentrations means that there exist sanctuary sites that are not or only partially penetrated by drugs. Therefore, reproductive fitnesses of cells depend not only on their cell types but also on their spatial locations, leading to a rugged fitness landscape (since drug concentrations in different spatial compartments are not necessarily continuous but discrete in space). As shown in previous studies [11, 14, 15], if the fitness of resistant cells as compared to sensitive cells is neutral or even slightly advantageous in the absence of drugs, the acquisition of resistance not only becomes highly likely but also is accelerated, since there is no selection pressure against resistance. However, it is less clear about the most likely pathway to select for resistance, if resistance mutations incur a fitness cost in the absence of drugs while conferring an advantage over sensitive cells in the presence of drugs. Recent mathematical modeling with laboratory test using mice suggests that resistance carries a fitness cost [68]. In view of this, the present study is focused on the latter scenario with fitness cost of resistance, although our approach works for any fitness landscape. We show that resistant mutants are unlikely to emerge *in situ* in compartments of high drug concentrations, but arise through the mutation-migration pathway; namely, metastatic cells acquire costly resistance in the sanctuary sites preceding migrating to and thriving in harsh compartments containing high levels of drugs.

## Results

**Fig 1:**
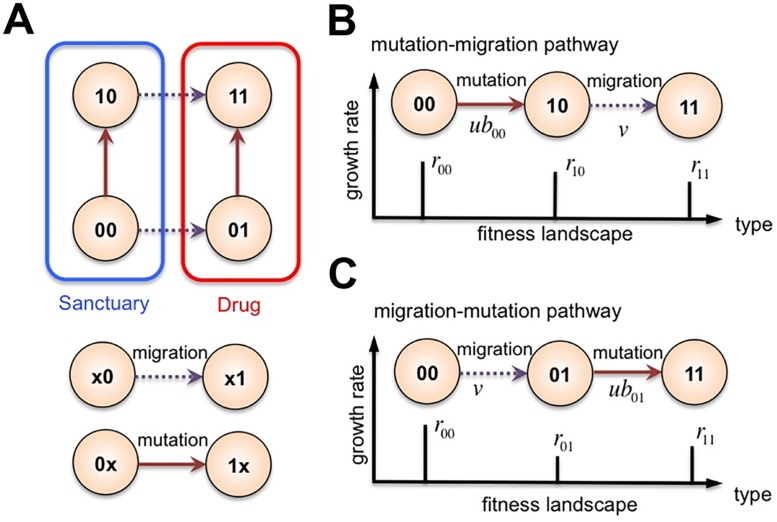
Schematic of the simple model. Here we study the scenarios where drug concentration and the rate of cell proliferation can be spatially dependent on the tumor microenvironment and *de novo* resistance mutations are needed to escape potent treatments (*i.e.*, targeted combination therapies). (**A**) Even if only a small number of cells reside in the sanctuary and/or are slowly replicating, they persistently seed the non-sanctuary compartment that would have almost certainly become void otherwise. Therefore, resistance in the non-sanctuary compartment arises through two competing pathways: (**B**) the “migration-mutation” pathway in which sensitive cells first migrate to the harsh drug-containing compartment and then evolve resistance *in situ*, or (**C**) the “mutation-migration” pathway in which sensitive cells first acquire the resistant mutation in the sanctuary compartment and then migrate into and subsequently populate the drug-present compartment. We show that, under a wide variety of conditions, resistant cells thriving in compartments with high levels of drugs are likely to originate from sanctuary sites, where they acquired resistance preceding migration.

Without loss of generality, let us first study the simplest possible ‘drug-sanctuary’ scenario for treatment failure due to imperfect drug penetration, as illustrated in Fig. 1. We consider two spatial compartments with dichotomic distributions of drugs: compartment ‘0’ can hardly be penetrated by the cancer drug, thereby representing a perfect drug sanctuary site; compartment ‘1’ is distributed with an adequate amount of drugs that is able to completely wipe out any wild type cells. We assume that one point mutation is sufficient to confer high levels of resistance to the maximum possible concentration of drugs administered during therapy. We denote the genotypes of cells by the number of acquired point mutations: the wild type ‘0’ and the resistant type ‘1’ (see Materials & Methods for a detailed description of the model). This minimal model, albeit overly simplified, offers intuitive insights into understanding how spatial drug heterogeneity facilitates the acquisition of *de novo* resistance during cancer therapies. Later on, we will extend this simple model to more realistic cases with multiple cell types and with multiple compartments, where multiple point mutations can successively accrue to confer resistance to exceedingly high drug concentrations [54].

It is well known that cancer relapse strongly depends on the initial size of the tumor or metastases at the starting of treatment [11, 14–17, 64, 67, 69, 70]. Without teasing out the effect of population size, we cannot clearly pinpoint the role of spatial drug heterogeneity in tumor resistance. Thus, let us start with our theoretical analysis with tracking the lineages derived from a single sensitive cell that can be initially placed in either compartment. Nevertheless, using the multiplicative properties of branching processes, we can calculate the probability of escape and the (conditional) average time to resistance for any given initial conditions of tumor size or metastases (see derivation details in S1 Text).

The sanctuary compartment provides far more favorable condition for breeding resistance than the drug-containing compartment (see comparisons of the probabilities of drug-environment-dependent escape in S1 Text). In most circumstances, it is the sanctuary compartment that persistently seeds the drug-containing compartment, which would have almost certainly become void otherwise due to effective levels of potent drugs. For this reason, in the presence of a sanctuary compartment, there exist two competing pathways to lead to the outgrowth of resistance in the compartment of high drug concentrations, as depicted in Fig. 1B and 1C. One is the “migration-mutation” pathway: sensitive cells first emigrate from the sanctuary compartment and then adapt *in situ* to the non-sanctuary compartment with high drug concentrations. The other is the “mutation-migration” pathway: sensitive cells first mutate and acquire resistance in the sanctuary compartment, and then migrate to and populate the compartment with high drug concentrations. To understand how the presence of tumor sanctuary sites and cell migration together affect the resistance evolution, we need to determine which pathway provides the more likely path to resistance.

Because cells of the same types have different fitness in the two compartments, we regard migration as a sort of status change in spatial locations. In this way, the two competing pathways can be seen as three-type branching processes, respectively, with different fitness landscapes. Our results are based on multi-type branching processes (Fig. 1B and 1C) [17, 64, 71–73] (see derivation details in S1 Text). To make progress, let us assume the following rugged fitness landscape owing to the heterogeneity of drug distributions across compartments. In the sanctuary compartment 0, both sensitive and resistant cells have supercritical replication potential, but resistant cells have slightly lower replication rates than sensitive type due to the cost of the resistant mutation; that is, *b*_00_ > *d*_00_, *b*_10_ > *d*_10_, and *b*_10_ < *b*_00_. In the non-sanctuary compartment 1, sensitive cells have a subcritical replication potential while resistant cells still have a supercritical replication potential; that is, *b*_01_ < *d*_01_, *b*_11_ > *d*_11_, and *b*_11_ > *b*_01_.

**Fig 2:**
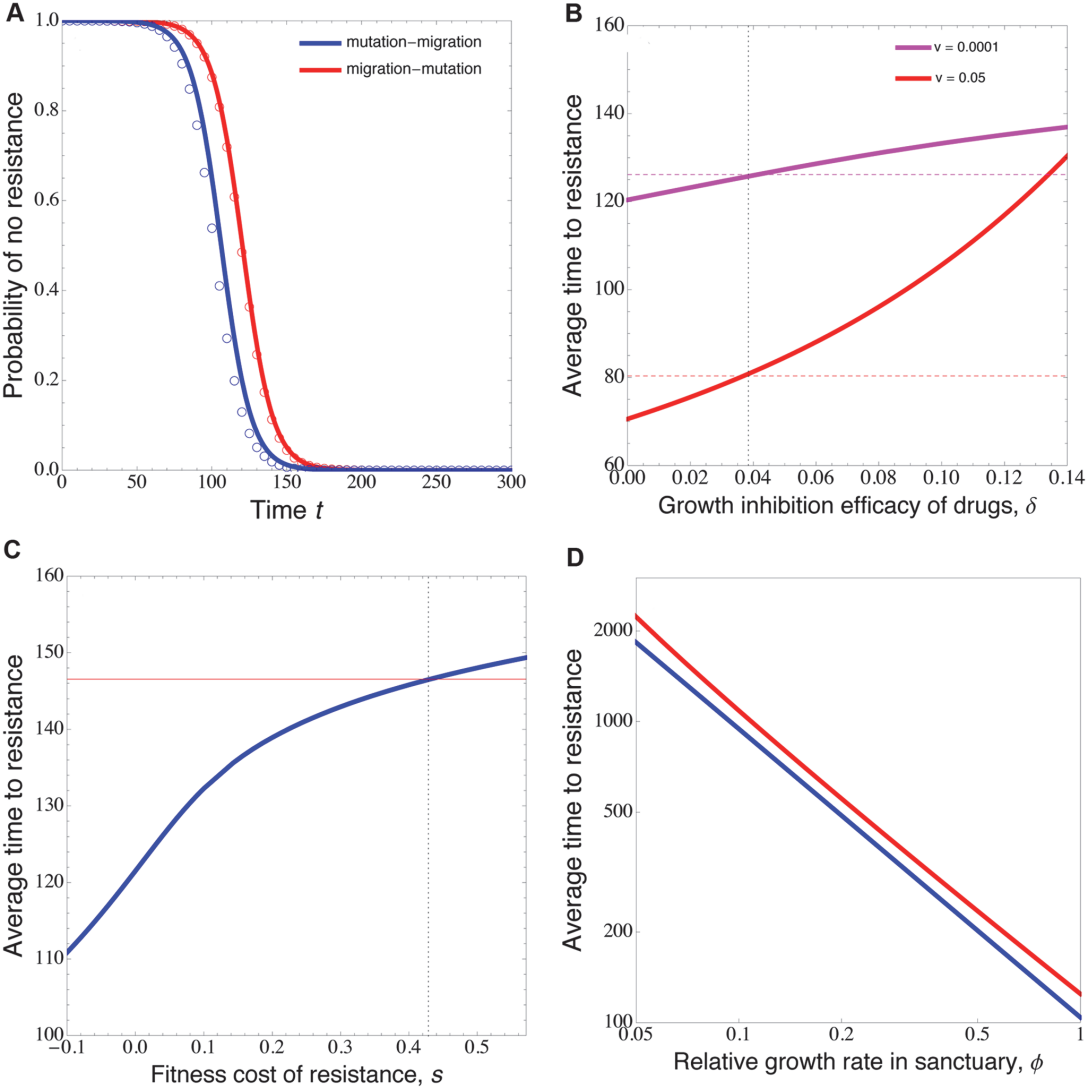
Competing pathways to selection for resistance. (**A**) Shown is the probability that no resistant cells are present at time *t*, conditional on non-extinction, for the two respective pathways. Circles are closed-form approximations, and solid lines are obtained by numerically solving the differential equations of the probability generating functions (see S1 Text for details). In (**B**), the solid lines show the average time to resistance (conditional on non-extinction), following the migration-mutation pathway, as a function of the drug efficacy of growth inhibition of sensitive cells, *δ*, and with two values of the migration rate, *v*. The dashed horizontal lines are the average time to resistance following the mutation-migration pathway. The vertical line marks the theoretical critical *δ* = *s*/(1+*s*), above which the mutation-migration pathway is faster. (**C**) plots the average time to resistance, following the mutation-migration pathway, as a function of the fitness cost *s* of resistance in the absence of drugs. Resistant mutations are neutral or advantageous for *s* ≤ 0, while being costly for *s* > 0. The horizontal line is the average time to resistance following the migration-mutation pathway. The dotted vertical line marks the critical *s* value, expressed in terms of *δ*, *s* = *δ*/(1−*δ*). (**D**) shows the dependence of the average time to resistance on the relative growth rate *r* of sensitive cells in the sanctuary versus in the non-sanctuary compartment. The scaling parameter *r* ∈ (0, 1] controls the degree to which cells in the sanctuary grow slower than in the non-sanctuary compartment due to the differences in microenvironment. Reducing the growth rate of cells in the sanctuary prolongs the time to resistance and equally affects the two pathways. Parameters: (**A** - **D**) *b*_11_ = 0.45, *d*_01_ = *d*_11_ = 0.4, *u* = 10^−4^; (**A** - **C**) *b*_00_ = 0.5, *d*_00_ = *d*_10_ = 0.4; (**C** - **D**) *b*_01_ = 0.35; (**A**) *b*_01_ = 0.39, *b*_10_ = 0.48, *v* = 10^−3^; (**B**) *b*_01_ = *b*_00_(1−*δ*), *s* = 0.04, *v* = 10^−4^, 0.05; (**C**) *b*_10_ = *b*_00_(1−*s*), *v* = 10^−4^; (**D**) *b*_00_ = 0.5*ϕ*, *d*_00_ = 0.4*ϕ*, *s* = 0.01, *v* = 10^−3^.

Although our method works for any mutation rate and migration rate (see Materials & Methods), we find a simple condition in the limit of low rates of mutation and migration (Fig. 2). (Such limiting results should hold true in realistic settings of cancer dynamics, given that the point mutation rate of most cancers is estimated to be 10^−8^ ∼ 10^−9^ [1, 2] and the dissemination rate of pancreatic cancer cells ∼ 10^−7^ [74].) That is, the mutation-migration pathway is faster than the counterpart, the migration-mutation pathway, to result in resistance in the drug-containing compartment, if the fitness landscape satisfies the following inequality:

$$\begin{array}{c} \frac{b_{00}}{b_{00}-d_{00}-\left(b_{10}-d_{10}\right)}>\frac{b_{01}}{b_{00}-d_{00}-\left(b_{01}-d_{01}\right)}. \end{array}$$ (1)

Since most targeted therapies have cytostatic effects, other than the cytotoxic effects commonly seen in traditional chemotherapy [75], in this work we assume the drug inhibits cell proliferation and does not affect death rates of cells. The condition above can be greatly simplified when the two compartments provide exactly the same condition for population outgrowth in the absence of drugs. The rugged fitness landscape due to spatial drug heterogeneity, therefore, can be reflected solely by differences in proliferation rates (i.e., *d*_00_ = *d*_10_ = *d*_01_ = *d*_11_). Moreover, the fitness cost of resistance, *s*, for resistant cells located in the sanctuary compartment 0 can be parameterized as *b*_10_ = (1−*s*)*b*_00_, while the fitness cost of sensitivity, *δ*, for wild type cells in the drug-containing compartment 1, *b*_01_ = (1−*δ*)*b*_00_. Substituting these parameterizations into (1), we arrive at the much simplified condition in terms of *s* and *δ*:

$$\begin{array}{c} \delta >\frac{s}{1+s}. \end{array}$$ (2)

We immediately observe that this condition is expected to be fulfilled in most cases since potent therapy should be characterized by *δ* > *s*.

Our mathematical framework allows us to calculate the probability of no resistance with respect to time, conditional on non-extinction and starting with a single sensitive cell following each pathway separately (Fig. 2A). We also calculate and compare the average time to resistance (relapse time conditional on non-extinction) following each pathway, as shown in Fig. 2B. The result demonstrates that the simple condition as given in Eq. (2) works well in the limit of low migration rate and remains a good approximation for intermediate migration rates. Moreover, if resistant mutation is neutral or even advantageous in the sanctuary, then the mutation-migration pathway is always much faster than the migration-mutation pathway to result in resistance in the drug-containing compartment (Fig. 2C).

Another important aspect of tumor microenvironment is characterized by differential rates of proliferation across compartments in the absence of treatment: cells in the sanctuary compartment may have slower replication and turnover rates than these in other compartments [22]. We thus use the parameter 0<*ϕ* ≤ 1 to rescale the proliferation and death rates of cells in the sanctuary compartment 0 relative to that in the drug-containing compartment 1: *d*_00_/*ϕ* = *d*_10_/*ϕ* = *d*_01_ and *b*_01_ = (1−*δ*)*b*_00_/*ϕ* (which implies that in the absence of treatment, cells grow 1/*ϕ* times as fast in compartment 1 as when located in compartment 0). Although one should refer to the general inequality (1) as the exact condition for the mutation-migration pathway to be predominant, simple algebra shows that the simplified inequality (2) is still a necessary condition in this case. In fact, reducing the rate of cell proliferation in the sanctuary equally affects the two pathways, delaying the time to resistance (Fig. 2D). Taken togethers, these results demonstrate that, under a wide variety of conditions (including the ranges of parameter values relevant to cancer), prevailing resistant cells in compartments with high levels of drugs are likely to originate from sanctuary sites, where they acquired resistance preceding migration.

**Fig 3:**
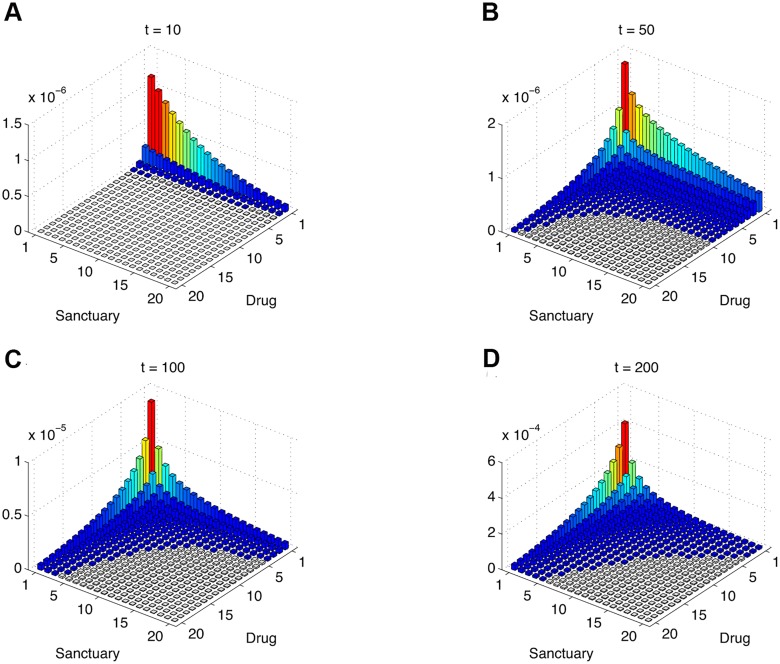
Spatio-temporal snapshots of emerging drug resistance. Panels (**A**) - (**D**) plot the joint probability density of the numbers of resistant cells in both compartments at time points *t* = 10, 50, 100, 200, respectively, starting with a single sensitive cell placed in the non-sanctuary compartment. Because the tumor microenvironment mediates the rate of cell proliferation, we assume that resistant cells grow much faster in the non-sanctuary compartment than in the sanctuary compartment. Panels (**A**) and (**B**) show that evolution of resistance *in situ* in the non-sanctuary compartment is unlikely, and that the sanctuary compartment provides an escape hatch for sensitive cells originally in the non-sanctuary compartment to breed resistance. Panels (**C**) and (**D**) show that it becomes increasingly likely that not only resistance gets established in the non-sanctuary compartment due to the constant seeding of resistant cells from the sanctuary compartment, but also its abundance quickly outnumbers that in the sanctuary. Parameters: *b*_00_ = 0.1, *d*_00_ = 0.05, *b*_01_ = 0.38, *d*_01_ = 0.4, *b*_10_ = 0.099, *d*_10_ = 0.05, *b*_11_ = 0.5, *d*_11_ = 0.4, *u* = 10^−4^, *v* = 10^−2^.

Of interest is to observe the evolutionary process initiated by a single sensitive cell located in the compartment of high drug concentration. We develop a numerical method to show the spatio-temporal dynamics of emerging drug resistance across spatial compartments (see Materials & Methods). Note that different from the constrained pathways analyzed in Fig. 2, in this case, migration is allowed to be bi-directional, and both pathways can be at work at the same time. Doing this enables us to study the emergence of resistance in a more natural and realistic setting. Fig. 3 shows the joint *probability* distribution of the numbers of resistant cells in the two compartments with respect to time. The skewed distribution in Fig. 3A suggests that the sanctuary compartment provides much more favorable condition to evolve resistance than the drug-containing compartment, and thus escaping from the drug-containing compartment to the sanctuary compartment is crucial to this end. Constantly seeding the drug-containing compartment with evolved resistant cells tends to make the distribution more balanced (cf Fig. 3B and 3C). As a result, resistance soon gets established in the drug-containing compartment, and its growth outpaces that in the sanctuary compartment (Fig. 3D), although the chance of having resistance increases steadily with time in both compartments. Similar results are obtained using different initial conditions, despite that resistance evolution is more likely (and sooner) to occur when the sensitive cell is initially placed in the sanctuary compartment than in the drug-containing compartment (cf. S1 Fig. and Fig. 3). Our results demonstrate that the sanctuary compartment, even though cells are slowly replicating therein, serves as an escape hatch, and indeed is most likely to be the breeding ground of resistance. Therefore, in the presence of sanctuary sites, the overwhelming outgrowth of resistance in the drug-containing compartment is an inevitable outcome due to the mutation-migration pathway.

**Fig 4:**
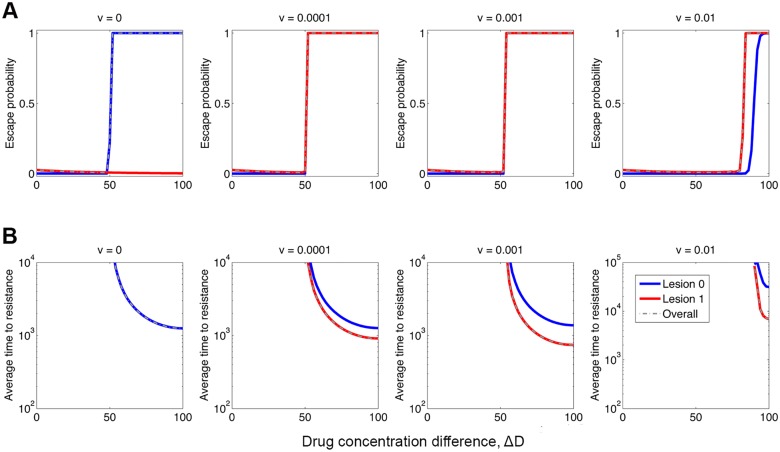
Outcomes of *in silico* treatment to eradicate metastases. The upper row panels (**A**) show the overall escape probabilities of the two metastatic lesions (dash-dotted curves), and that of the respective each lesion (solid curves), with respect to the increasing difference of drug concentration between the two metastatic compartments, Δ*D* = *D*_1_−*D*_0_, and for varying migration rates, *v*. Corresponding to (**A**), the lower row panels (**B**) show the average time to resistance (conditional on non-extinction), as a function of the level of heterogeneity in drug concentrations, Δ*D*. When treatment starts, a hypothetical cancer patient has two metastatic lesions: lesion 0 has *N*_0_ = 10^3^ cells and lesion 1 has *N*_1_ = 10^8^ cells. We also assume that cells in metastatic compartment 0 (*β*_0_ = 0.05, *α*_0_ = 0.04) grow much slower than in compartment 1 (*β*_1_ = 0.5, *α*_1_ = 0.4), in the absence of drugs. We assume the drugs have poorer penetration of lesion 0 than of lesion 1, and vary the difference of drug concentration from 0 (*D*_0_ = *D*_1_ = 50) to 100 (*D*_0_ = 0, *D*_1_ = 100). In this simulated case, only one point mutation is needed to confer strong resistance to high drug concentrations. Parameters: IC_50_ = 50, *m* = 2, *D̄* = (*D*_0_+*D*_1_)/2 = 50, *s* = 0.01, *ρ* = 5, *u* = 10^−9^, *v* = 0, 10^−4^, 10^−3^, 10^−2^.

Having illuminated the essence of the problem (Fig. 1–3), we now turn to predict outcomes of hypothetical treatments to eradicate (two) metastases in cancer patients (Fig. 4). We assume the total mass of metastasis is relatively small so that treatment starts without any pre-existing resistance. In particular, we assume that the two metastatic lesions differ in size as well as in the level of drug penetration, because of different microenvironments. Specifically, cells in lesion 0 grow much more slowly than in lesion 1 in the absence of drugs, yet the drugs have better penetration of lesion 1 than of lesion 0. To this end, we continuously vary the difference of drug concentrations in the two compartments, Δ*D* = *D*_1_−*D*_0_, while keeping the total sum of concentrations constant. Let us now specifically incorporate into the simple model a Hill function that describes concentration-dependent killing efficacy of drugs (see Materials & Methods).

Without migration and under homogeneous drug concentrations the metastatic cancer almost certainly can be eradicated successfully. However, a worrying situation arises in the presence of a sanctuary with sufficiently low drug concentrations. With increasing difference of drug concentration between the two metastatic compartments, treatment responses exhibit a sharp transition from successful eradication to failed treatment due to acquired resistance (Fig. 4A). Our mathematical framework allows us to calculate the relapse curve, 1−*p*_*s*_(*t*), where *p*_*s*_(*t*) is the probability of no resistance by time *t* following treatment, and the (conditional) average time to patient relapse due to acquired resistance (see Materials & Methods). Fig. 4B shows that the relapse is accelerated by the spatial heterogeneity; the larger Δ*D*, the faster the relapse. In this simulated hypothetical patient with small lesions, the worst-case scenario is when the drug cannot penetrate lesion 0 at all, and thus the relapse occurs on average approximately 10^3^ days. The relapse could have occurred within weeks if the treatment starts with much bigger lesions (S2 Fig.).

Especially when cells can migrate, resistance results not only from cells originally in the metastatic compartment 0 but also from these escaping from compartment 1. Noteworthy, cell lineages originating from lesion 1 are faster to evolve resistance than these originating from lesion 0 (Fig. 4B). This result is mainly due to the initial condition used: lesion 1 is much larger than lesion 0 that the influx of escaping cells to the sanctuary exceeds the number of cells *in situ*. Since we are considering a branching process, larger population size is more likely to generate resistant mutation. Indeed, as shown in S2 Fig., if the size of lesion 1 is smaller, cells lineages originating from lesion 1 are actually slower to evolve resistance than from lesion 0.

We emphasize that the monotonic decreasing relationship of relapse time with increasing the drug concentration difference, Δ*D*, is due to: (1) one point mutation is sufficient to confer strong resistance to the maximum possible concentration in compartment 1 (*D*_1_ = 100) in this simulated case; (2) compartment 0, which is distributed with less and less amount of drugs with increasing Δ*D*, provides an increasingly favorable condition for the evolution of resistance and thus renders shorter relapse time, since the mutation-migration pathway is the most contingent pathway for resistance evolution as shown before. Under different assumptions of fitness effects of mutations as described in Eq. (3), however, multiple point mutations might be required to confer sufficient level of resistance to increasingly high concentrations in compartment 1, as Δ*D* increases. This variation does not change the general picture about how the presence of sanctuary sites impairs the effectiveness of cancer therapies (S3A Fig.), but the time to the *sufficient* levels of resistance may well depend on how many point mutations are needed to this end and exhibits an abrupt increase when Δ*D* is increased beyond a critical threshold value (the vertical line in S3B Fig.). For very large Δ*D*, only two-point mutants are able to survive in compartment 1, wherein the abundances of one-point mutants and sensitive cells are maintained by the ‘immigration-death’ dynamics of the branching process. As time passes by, two-point mutants eventually pop up, most likely in the sanctuary compartment 0, and subsequently immigrate to and populate the compartment 1.

Taken together, Fig. 4 demonstrates that metapopulation dynamics arising from migration and seeding, together with the presence of sanctuary sites, play an important role in the rapid emergence of resistance. Furthermore, only for migration rates below a certain critical threshold does the spatial heterogeneity in drug concentrations speed up the emergence of resistance. Excessively high migration rates actually slow down resistance emergence (S4 Fig.), as the role of compartment structure is diminished by frequent migrations and consequently cells are exposed to the non-sanctuary compartment more often. It is worth noting that there exists an optimum migration rate that leads to the fastest emergence of resistance and that excessively high migration rates actually deter and delay the evolution of resistance (S4 Fig.). The results suggest that it may be helpful to improve clinical outcomes by combining targeted therapy with anti-metastatic treatment aimed at inhibiting cell motility as well as by enhancing drug transportation and distribution throughout all metastatic compartments.

We also extend the simple model to more general cases with multiple cell types and with multiple compartments (see detailed mathematical description in S1 Text). In this extended model, multiple point mutations can consecutively accrue to confer resistance to increasingly high drug concentrations. In particular, we study and compare the impact of the two different schemes of cell migration, local versus global migration, on the evolution of resistance (S5 and S6 Fig.). The numerical results confirm that our conclusions derived from the simple model above remain qualitatively unchanged. Additionally, we observe that strong resistance evolves much faster for local migration than global migration, particularly for sensitive cells located at the sanctuary sites with low drug concentrations (S5 Fig.). In other words, sequential local migration over spatial gradient of drug concentration following newly accrued point mutations (i.e., the mutation-migration pathway) plays an important part in leading to rapid selection for high-level resistance. This may be of particular relevance for the evolution of resistance within a solid tumor [22, 54]. Furthermore, apart from oversimplified migration schemes addressed in the current study, this full model can be readily extendedcenter to integrate with a realistic vascular network that regulates the metastatic routes of circulating tumor cells among target organs [53].

**Fig 5:**
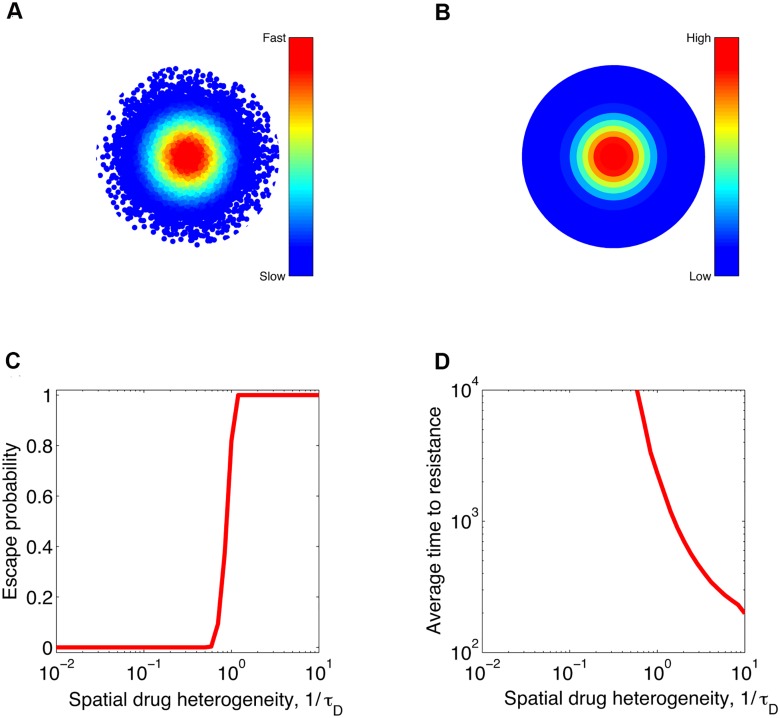
Tumor microenvironment facilitates the emergence of high levels of drug resistance. For simplicity, we specify the microenvironment of tumor cells in relation to their distance to the nearest blood vessel, *x*. Shown in (**A**) is the schematic section view of an “onion-structured” solid tumor with the nearest blood vessel located in the center. In the absence of drugs, both proliferation and turnover rates of cancer cells decrease with *x* [22]: birth rate *β*(*x*) = *b*_0_exp(−*x*/*τ*_*g*_) and death rate *α*(*x*) = *d*_0_exp(−*x*/*τ*_*g*_), where the parameter *τ*_*g*_ is the characteristic length scale of spatial decay in proliferation and turnover rates. Similarly, the spatial density distribution of tumor cells is assumed to exponentially decay with *x* and proportional to exp(−*x*/*τ*_*c*_), where *τ*_*c*_ is the characteristic length scale of decrease in cell density. Panel (**B**) plots the spatial drug gradient mediated by the tumor microenvironment [22]: *D*(*x*) = *D*_0_exp(−*x*/*τ*_*D*_), where *D*_0_ is the maximum possible concentration in the center and *τ*_*D*_ is the characteristic length scale of spatial decay of drug concentrations with respect to the distance to the blood vessel, *x*. Panels (**C**) and (D) show the escape probability and the average time to resistance (conditional relapse time to acquisition of two point mutations) as a function of the level of spatial drug heterogeneity, 1/*τ*_*D*_. Using a series of consecutive concentric circles with equal interval in between, we artificially divide the tumor into *M* compartments (similar to contour lines shown in **b**). Cells in the same compartment are regarded as homogeneous subpopulations. Tumor cells can migrate to the two nearest neighbouring compartments with equal probability *v*/2. Acquisition of two point mutations is needed to survive in the center with the maximum drug concentration, *D*_0_. Parameters: tumor size *N* = 10^11^, *τ*_*g*_ = *τ*_*c*_ = 1cm, *D*_0_ = 500, *b*_0_ = 0.5, *d*_0_ = 0.4, *M* = 30, *n* = 3, IC_50_ = 50, *m* = 2, *s* = 0.01, *ρ* = 5, *u* = 10^−9^, *v* = 2×10^−4^.

Last but not least, let us demonstrate how the evolution of resistance can be facilitated by the microenvironment within a tumor on the microscopic level [22]. Specifically, we consider a solid tumor consisting of 10^11^ cells which is about 4.6 cm in diameter [57]. Fig. 5A shows the schematic representation of tumor microenvironment of cells surrounding a blood vessel located in the center: the rate of proliferation of tumor cells decreases with increasing distance from the central blood vessel in the absence of treatment. Similarly, the delivery of cancer drugs is also compromised in the presence of treatment, thereby resulting in the spatial drug gradient as illustrated in Fig. 5B. The level of spatial drug heterogeneity is represented by 1/*τ*_*D*_: the larger of this value, the more poorly the drug penetrates distal tumor issues away from the nearest blood vessel. To make progress in our calculations, we artificially divide the tumor into *M* = 30 spatial compartments with consecutive concentric circles with equal interval in between from center to surface. We confirm that dividing more compartments leads to almost the same results as shown here in Fig. 5C and 5D. In this simulated example, two point mutations are required to confer full resistance to the maximum drug concentration in the center. As shown in Fig. 5C, the tumor can be eradicated under perfect drug penetration where the drug is almost homogeneously distributed throughout the entire tumor population (extremely small 1/*τ*_*D*_). In contrast, inadequate penetration of the tumor gives rise to sanctuary sites, these outer compartments that are most distant away from the blood vessel and thus exposed with the least amount of drugs. Therefore, cancer therapy fails certainly with large values of 1/*τ*_*D*_. In line with Fig. 4, relapse occurs sooner with increasing spatial drug heterogeneity, 1/*τ*_*D*_ (Fig. 5D). Moreover, distal cells, although slowly proliferating, are more likely to generate resistance than these proximal cells that are affected most by the drug. These results quantitatively demonstrate that tumor microenvironment mediates cell sensitivity to drugs and thus plays an important role in drug resistance acquired *during* treatments [22, 29, 54].

## Discussion

Here we study the roles that cell motility and spatial heterogeneity in drug concentrations play in the emergence of acquired resistance to cancer therapy. Cancer cells can migrate from one spatial compartment to another. As compartments may contain different levels of drugs, the cells experience distinct selection pressure for resistance in different compartments. We calculate the probability of resistance and the average time to resistance for sensitive cells originally located in different compartments. We show that the presence of sanctuary sites with poor drug penetration can speed up the emergence of acquired resistance to cancer therapy. Moreover, we show that resistance is unlikely to arise *in situ* within high concentration compartments, but rather that resistance first emerges in sanctuary sites and then spreads to and populates other compartments with high concentrations that would be able to completely inhibit growth of sensitive cells. This result is in line with a prior analysis of competing pathways to resistance, based on an ecological source-sink model with logistic population growth [76]. In spite of that, our study is specifically aimed at understanding the role of tumor microenvironment in the evolution of resistance during treatment.

Previous studies both empirically and theoretically reveal that spatial drug gradient can facilitate the evolution of antibiotic resistance [25–28]. Although well suited for studying experimental microbial evolution [26, 27], these models are not directly applicable to the context of cancer; especially solid tumours with intrinsic heterogeneity in their microenvironments warrant a thorough separate investigation [18–20]. Furthermore, in these prior models [26, 27], compartments are placed in an order with increasing drug concentration, sequential migration occurs only between the two nearest neighbor compartments, and evolution begins within the sanctuary while other compartments are initially void. In contrast, the present mathematical framework takes into account realistic concentration-dependent response for any initial population of cancer cells distributed over multiple metastatic compartments, and allows us to calculate the risk of acquiring resistance as well as to ascertain the timing of relapse. We note that it is promising for future studies to improve mathematical tractability of this problem addressed in this work, for example, by using various analytical techniques as detailed in Refs. [69, 70]. Moreover, our model can be readily extended to incorporate specific migration schemes of cancer cells, *e.g.*, along with a more realistically connected vasculature [53]. In parallel, it is worth mentioning that the approach of using partial differential equations to describe spatio-temporal dynamics of cancer evolution [77–79] sheds a different yet useful light on the selection of resistance under cancer therapies [80, 81]. Taken together, our theoretical results improve our understanding of how metastatic cells can acquire resistance during treatment, especially in the presence of sanctuary sites.

In this study we focus on the evolution of resistance exclusively in the population of metastatic cells that have the same capacity to migrate. It has been shown that migratory cells, though with lower growth potential than non-migratory cells, can be selected for during therapy [82]. Extending this prior result, our results show that cell motility and the presence of sanctuary sites with little drug exposure are essential for the rapid acquisition of resistance by metastatic cells. Moreover, only for low migration rates below a certain threshold does spatial heterogeneity in drug concentration speed up resistance evolution. Arguably, this finding may help to explain qualitatively the differences of clinical successes in treating liquid cancer (such as chronic myeloid leukemia [7]) and solid tumors (such as melanoma [10]). Because of limited cell motility, metastases of solid tumor tend to have well-defined spatial compartments (namely, distal lesions), and thus are more prone to drug penetration problems. For this reason, additional attention should be paid to eliminate the sanctuary sites for cancer therapy [21].

In the current work, we only consider resistance to a single drug, or more precisely, to treatments with a drug or drug combination to which resistance can be generated by the accumulative acquisition of a set of point mutations. It is promising for future work to study multi-drug resistance requiring multiple sets of resistant mutations, given that combination therapy is increasingly used in clinical setting [83, 84]. (We refer to Ref. [85] for a most recent development in this matter with a focus on multi-drug resistance to antiviral combination treatments.) In addition to the spatial heterogeneity in drug concentrations addressed here, we think that epistatic interactions of resistant mutations to each drug may also be important and deserve further investigation [86].

In general, our work provides a mathematical and computational framework for studying how various aspects of tumor microenvironment, and spatial drug heterogeneity in particular, influence cell sensitivity to drugs and thus mediate the evolution of resistance *during* treatment. More important, our results may allow us to better understand the recent *in vitro* experiment that shows the rapid emergence of high-level resistance can be facilitated by the presence of spatial drug gradients and the motility of metastatic cancer cells along the gradient [54]. In this work, we demonstrate that the mutation-migration pathway is most relevant for establishing sufficient levels of resistance in areas of high drug concentrations that would have wiped out all initial sensitive cells. This theoretical result can stimulate further empirical research that aims at better understanding the spatio-temporal evolutionary dynamics of resistance (*e.g.*, the competing pathways of resistance as shown in Fig. 1B and 1C) using single-cell tracking and sequencing technologies.

We also show that the sanctuary sites are the most likely breeding ground for resistance and thus responsible for the widespread outgrowth of resistance in other compartments of high concentrations. This theoretical result can be extended to derive potential therapeutic strategies for increasing the efficacy of cancer therapy. Specifically, our results suggest that combining targeted therapy with anti-metastatic treatment might help improve clinical outcomes [87–89], especially when treating disseminated cancer. As demonstrated in the present work, inhibition of cell migration between compartments not only suppresses the escape route of sensitive metastatic cells to sanctuary sites and also prevents the dissemination of evolved metastatic cells from sanctuary sites to these compartments with high concentrations, where resistance is strongly selected for. In addition to targeting cell dissemination, it is desirable to enhance drug transportation and distribution throughout all metastatic compartments, in order to deter the rise of resistance [29].

The current model is minimalistic, but allows proof of principle. We leave out many important issues, such as cancer stem cells [90–93], cellular quiescence and cancer dormancy [94, 95], and the inefficiency of metastatic processes [96–98]. In particular, we have considered the dynamics of the evolution of drug resistance in a situation in which cells are able to move freely from one compartment (tumor) to another. This first approximation, while enlightening, may overestimate some of the dynamics that would occur in a more realistically connected vasculature, especially when the inefficiencies of metastasis, due to filtration and dissemination in the vascular network, are considered [52, 53]. With the increasing understanding of the molecular biology of metastasis as well as clinical advances in treating metastasis, we believe that it will become feasible to obtain accurate estimations of key parameters regarding metastatic burden (location and size) and metastatic rates. Then a calibrated model of this sort as introduced here can be used to simulate patient responses *in silico* and predict outcomes of treatments to eradicate the disseminated cancer [11], as well as to derive more efficacious treatment strategies, particularly for overcoming the problem of imperfect drug penetrations [29].

## Materials and Methods

### Minimal model

We focus the present study on the role that tumor microenvironment plays in the emergence of acquired resistance to potent cancer therapies, where *de novo* mutations are required to confer strong resistance to high drug concentrations. In our model, we explicitly account for the compartment structure of tumor microenvironment as well as the spatial heterogeneity in drug concentrations across compartments. For proof of concept, we focus on the simplest possible case with only two compartments and two types of cells in the main text. Without loss of generality, we assume that drugs have better access to compartment 1 than to compartment 0. In contrast to conventional chemotherapy agents that have cytotoxic effects, most molecularly targeted cancer therapies have cytostatic effects on cancer cells [75]. Moreover, it is commonly found that the efficacy of drugs is concentration-dependent in pharmacological kinetics studies ranging from antimicrobial treatment to cancer therapy [99–102]. Therefore, it is plausible for us to specifically consider concentration-dependent inhibition of cell replication in response to cancer therapy.

Denote by *D*_*i*_ the drug concentration in compartment *i*. Upon division, one of the daughter cells can mutate with probability *u* to become resistant. Denote by *i* the genotype of a cancer cell if it has acquired *i* point mutations (*i* = 0, 1). The fitness of a cell depends on its type and spatial location. Specifically, the replication rate of a cancer cell, *b*_*ij*_, is determined by its genotype *i* and spatial location *j* (*j* = 0, 1) as follows,

$$\begin{array}{c} b_{ij}=\beta_{j}\frac{1-is}{1+{\left[\frac{D_{j}}{\rho^{i}{\text{IC}}_{50}}\right]}^{m}}. \end{array}$$ (3)

Here we use a Hill function for the drug response curve [103]. *β*_*j*_ (*α*_*j*_, respectively) is the division rate (the death rate, respectively) of a sensitive cell located in compartment *j* in the absence of drugs, *s* is the cost of resistance per point mutation in the absence of drugs, IC_50_ is the drug concentration that is needed to inhibit cell growth by one half of its original rate, *ρ* is the fold increase in IC_50_ per mutation, and *m* determines the steepness of the Hill function. Previous studies have fitted empirical data to similar Hill functions as given above [Eq. (3)], to indicate antibiotic resistance and antiviral resistance to various treatment regimens [99, 103]. Although a full characterization of cancer drug resistance in this way has yet to be done, our general results are not dependent on specific parameter choices of *s*, *ρ*, *m* and IC_50_. The death rate of a cell with genotype *i* and in compartment *j* is unaffected by the presence of drugs and equal to that of sensitive cells irrespective of their genotypes, *d*_*ij*_ = *α*_*j*_. The net growth rate is denoted by *r*_*ij*_ = *b*_*ij*_−*d*_*ij*_. Cells can migrate between the two compartments with rate *v*. The unit of all rates is per cell per day.

### Generating function approach

Denote by *F*_*ij*_(**X**; *t*) the probability generating function for the lineages at time *t* initiated by a single *ij*-type cell, where **X** = [*x*_00_, *x*_01_, *x*_10_, *x*_11_]^*T*^ denotes the vector of dummy variables with elements *x*_*ij*_ representing each *ij*-type of cells. The backward equations for this branching process are (see S1 Text for how to derive them)

$$\begin{array}{ccc} \frac{\partial F_{00}}{\partial t} & = & d_{00}+b_{00}\left(1-u\right)F_{00}^{2}+b_{00}uF_{00}F_{10}+vF_{01}-\left(d_{00}+b_{00}+v\right)F_{00}\\ \frac{\partial F_{01}}{\partial t} & = & d_{01}+b_{01}\left(1-u\right)F_{01}^{2}+b_{01}uF_{01}F_{11}+vF_{00}-\left(d_{01}+b_{01}+v\right)F_{01}\\ \frac{\partial F_{10}}{\partial t} & = & d_{10}+b_{10}F_{10}^{2}+vF_{11}-\left(d_{10}+b_{10}+v\right)F_{10}\\ \frac{\partial F_{11}}{\partial t} & = & d_{11}+b_{11}F_{11}^{2}+vF_{10}-\left(d_{11}+b_{11}+v\right)F_{11}. \end{array}$$ (4)

The initial condition is given by *F*_*ij*_(**X**; 0) = *x*_*ij*_.

### Probability of acquired resistance

To extract marginal joint probabilities from generating functions, we use the Cauchy’s integral method to replace the task of taking multiple derivatives. Similar methods have been used in the literature [104, 105]. For example, the probability density of the number of resistant cells, *p*_*mn*_(*t*), in both compartments at time *t*, as shown in Fig. 3, is given by

$$\begin{array}{c} p_{mn}\left(t\right)={\left.\frac{1}{m!}\frac{1}{n!}\frac{\partial F_{01}\left(1,1,x,y;t\right)}{\partial x^{m}\partial y^{n}}\right|}_{x=0,y=0}. \end{array}$$

Using the Cauchy’s integral formula, we obtain

$$\begin{array}{ccc} p_{mn}\left(t\right) & = & \frac{1}{m!n!}\frac{m!n!}{{\left(2\pi i\right)}^{2}}\oint_{c}\oint_{c}\frac{F_{01}\left(1,1,x,y;t\right)}{x^{m+1}y^{n+1}}dxdy\\ & = & \frac{1}{4\pi^{2}}\int_{0}^{2\pi }\int_{0}^{2\pi }F_{01}\left(1,1,e^{i\theta_{1}},e^{i\theta_{2}};t\right)e^{-im\theta_{1}}e^{-in\theta_{2}}d\theta_{1}d\theta_{2} \end{array}$$ (5)

Applying the trapezoid rule to approximate the double integral, we arrive at:

$$\begin{array}{c} p_{mn}\left(t\right)\approx \frac{1}{N^{2}}\sum_{i_{1}=0}^{N-1}\sum_{i_{2}=0}^{N-1}F_{01}\left(1,1,e^{ii_{1}\frac{2\pi }{N}},e^{ii_{2}\frac{2\pi }{N}};t\right)e^{-imi_{1}\frac{2\pi }{N}}e^{-ini_{2}\frac{2\pi }{N}} \end{array}$$

Note that this formula above is essentially equivalent to the discrete Fourier transform of the generating function. To reduce the aliasing effect arising from spectral methods, we set *N* = 1000(≫ 20) for the results presented in Fig. 3.

### Conditional time to resistance

We use the conditional probability, *p*_*s*_(*t*), of having no resistant cells in both compartments to determine the average time to resistance starting with sensitive cells in either compartment. Specifically, starting with a single sensitive cell in compartment 1, the conditional probability *p*_*s*_(*t*) is given by

$$\begin{array}{c} p_{s}\left(t\right)=\frac{F_{00}\left(1,1,0,0;t\right)-F_{00}\left(1,1,0,0;\infty \right)}{1-F_{00}\left(1,1,0,0;\infty \right)}. \end{array}$$ (6)

Then average time to resistance *T̄*_*r*_ (relapse time) can be calculated as follows,

$$\begin{array}{c} {\bar{T}}_{r}=\int_{0}^{\infty }p_{s}\left(\tau \right)d\tau . \end{array}$$ (7)

### Full model

In S1 Text, we study more general cases where *n* point mutations are needed to confer full resistance and cancer cells can move between *M* compartments with restricted local or unrestricted global migration. Drugs are distributed over *M* spatial compartments according to given levels of spatial concentration heterogeneity. In particular, we consider two different schemes of migration: local migration versus global migration. Local migration means that compartments are situated on a “ring” where a cancer cell can only migrate to the two nearest neighbor compartments with equal probability *v*/2. In contrast, global migration means compartments are fully connected where a cancer cell is allowed to migrate from one compartment to any other one with equal probability *v*/(*M*−1). This extended model allows us to study how metastatic cancer cells acquire increasing levels of drug resistance as they migrate along a spatial gradient of drug concentration and thrive in areas of excessively high drug concentrations, as shown in a recent in vitro experiment [54].

## Supporting Information

S1 Fig Spatio-temporal snapshots of emerging drug resistance. Panels (**A**) - (**D**) plot the joint probability density distribution of the numbers of resistant cells in both compartments at time points *t* = 10, 50, 100, 200, respectively, starting with a single sensitive cell placed in the sanctuary compartment. Parameters are the same as in Fig. 3.
(TIF)

S2 Fig The average time to resistance as a function of the size of lesion 1, rescaled by the mutation rate, *uN*_1_. Relapse occurs sooner with bigger tumor size at the start of therapy. Relapse is destined to happen in the presence of drug sanctuary and cell motility. The situation is even worse for really big tumor sizes at the start of therapy; relapse can happen within weeks. Parameters: *D*_0_ = 0, *D*_1_ = 100, *v* = 10^−4^, and other parameters are the same as in Fig. 4.
(TIF)

S3 Fig The relapse time depends on the number of point mutations that is needed to confer sufficient levels of resistance to increasingly high drug concentrations. (**A**) shows the escape probability as a function of the difference in drug concentrations between the two metastatic compartments, Δ*D*. (**B**) shows how the average time to resistance changes with increasing Δ*D*. The vertical line marks the critical value of Δ*D* above which two point mutations are required to confer sufficient levels of resistance to increasingly high concentrations in compartment 1 while compartment 0 is the sanctuary containing lower level of drugs. Parameters: *ρ* = 3.5, *v* = 10^−4^, and other parameters are the same as in Fig. 4.
(TIF)

S4 Fig Optimal migration rate. Parameters: *D*_0_ = 0, *D*_1_ = 100, and other parameters are the same as in Fig. 4.
(TIF)

S5 Fig The role of cell motility in the emergence of full resistance under local and global migrations. Shown are the escape probabilities and the average conditional time to resistance for a single sensitive cell initially placed in each compartment, with increasing migration rate, *v*. The spatial heterogeneity in drug concentrations is realized by using a rescaled Normal distribution with a peak in the central compartment, and the level of heterogeneity is denoted by the standard deviation, *σ*, of the concentration distribution over compartments. Parameters: *M* = 20, *n* = 5, IC_50_ = 100, *m* = 2, *ρ* = 1.1, *D̄* = 50, *s* = 0.01, *b*_0_ = 0.2, *d*_0_ = 0.1, *μ* = 10^−4^, *σ* = 24.7.
(TIF)

S6 Fig The impact of the spatial heterogeneity in drug concentrations on the emergence of full resistance under local and global migrations. Shown are the escape probabilities and the average conditional time to resistance for a single sensitive cell initially placed in each compartment, with increasing spatial heterogeneity, *σ*. The spatial heterogeneity in drug concentrations is realized by using a rescaled Normal distribution with a peak in the central compartment, and the level of heterogeneity is denoted by the standard deviation, *σ*, of the concentration distribution over compartments. Parameters: *M* = 20, *n* = 5, IC_50_ = 100, *m* = 2, *ρ* = 1.1, *D̄* = 50, *s* = 0.01, *b*_0_ = 0.2, *d*_0_ = 0.1, *μ* = 10^−4^, *v* = 0.01.
(TIF)

S1 Text Supplementary Information for “Spatial heterogeneity in drug concentrations can facilitate the emergence of resistance to cancer therapy”. (PDF)
