# Supplementary figures and images for: Spatial Heterogeneity in Drug Concentrations Can Facilitate the Emergence of Resistance to Cancer Therapy
Source: PLoS Comput Biol. 2015 Mar 19;11(3):e1004142. doi: 10.1371/journal.pcbi.1004142 (PMC4366398; doi:10.1371/journal.pcbi.1004142)

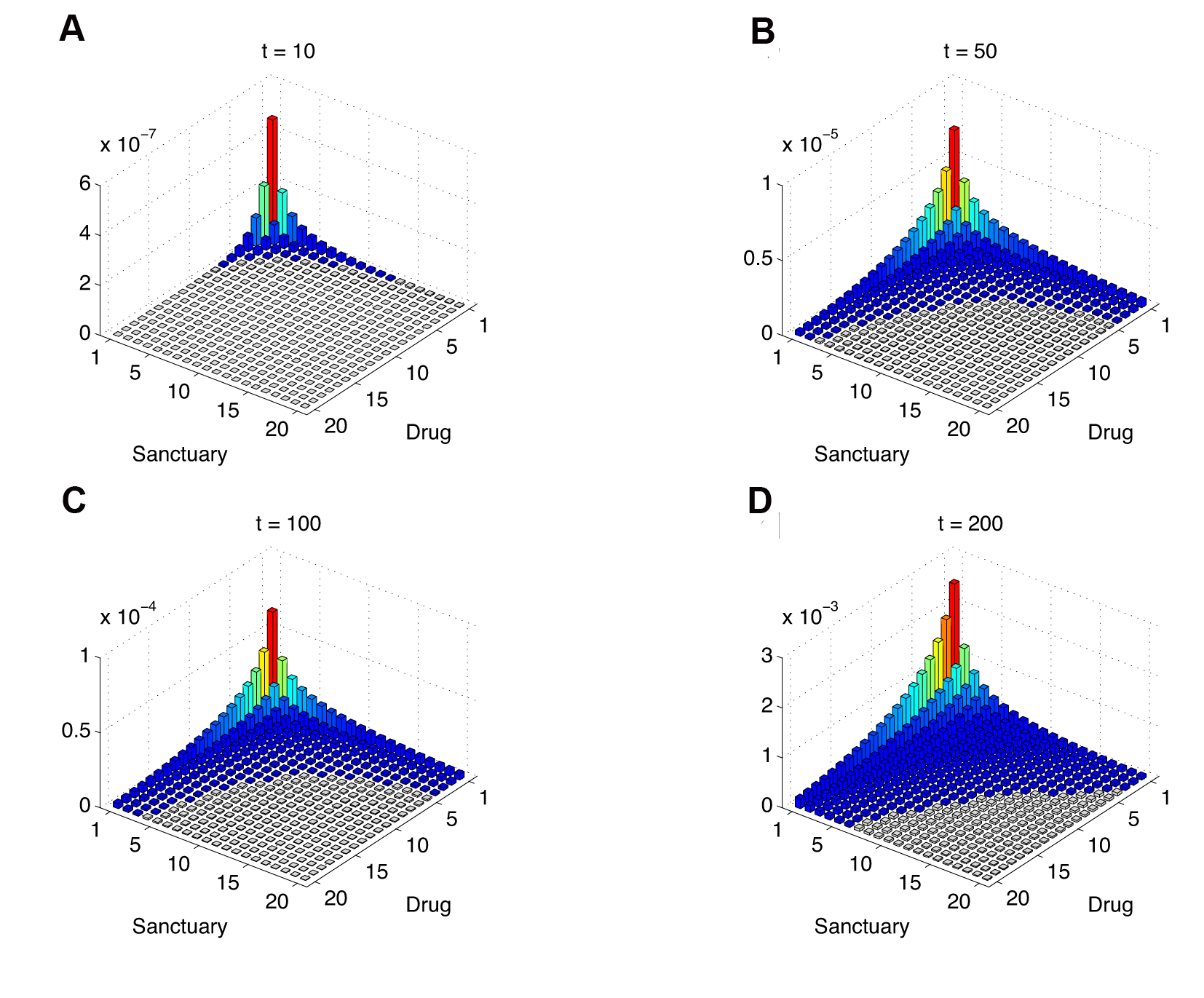

Supplement: S1 Fig — Panels (A) - (D) plot the joint probability density distribution of the numbers of resistant cells in both compartments at time points t = 10, 50, 100, 200, respectively, starting with a single sensitive cell placed in the sanctuary compartment. Parameters are the same as in Fig. 3. (TIF) [file pcbi.1004142.s001.tif]

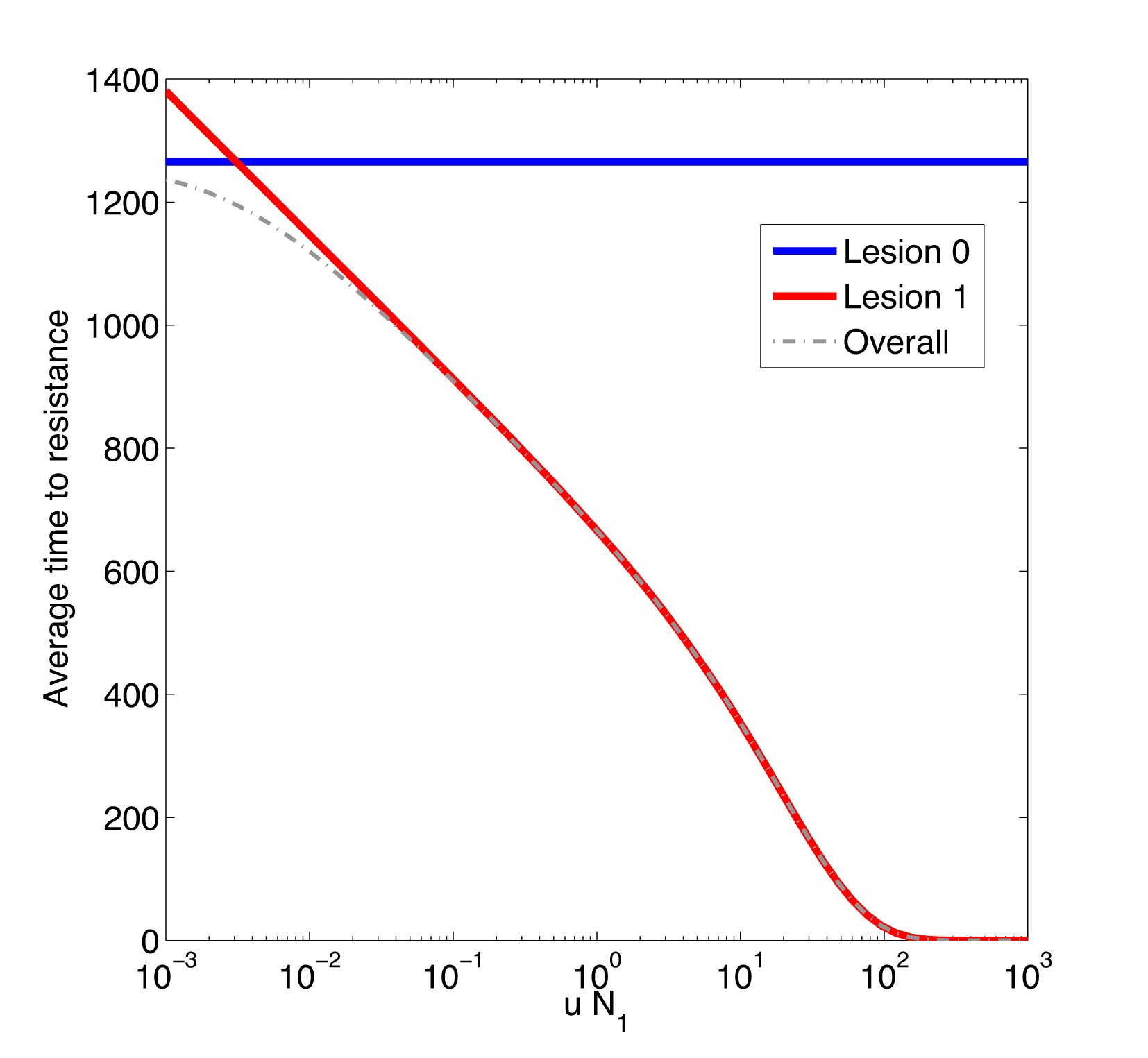

Supplement: S2 Fig — Relapse occurs sooner with bigger tumor size at the start of therapy. Relapse is destined to happen in the presence of drug sanctuary and cell motility. The situation is even worse for really big tumor sizes at the start of therapy; relapse can happen within weeks. Parameters: D 0 = 0, D 1 = 100, v = 10−4, and other parameters are the same as in Fig. 4. (TIF) [file pcbi.1004142.s002.tif]

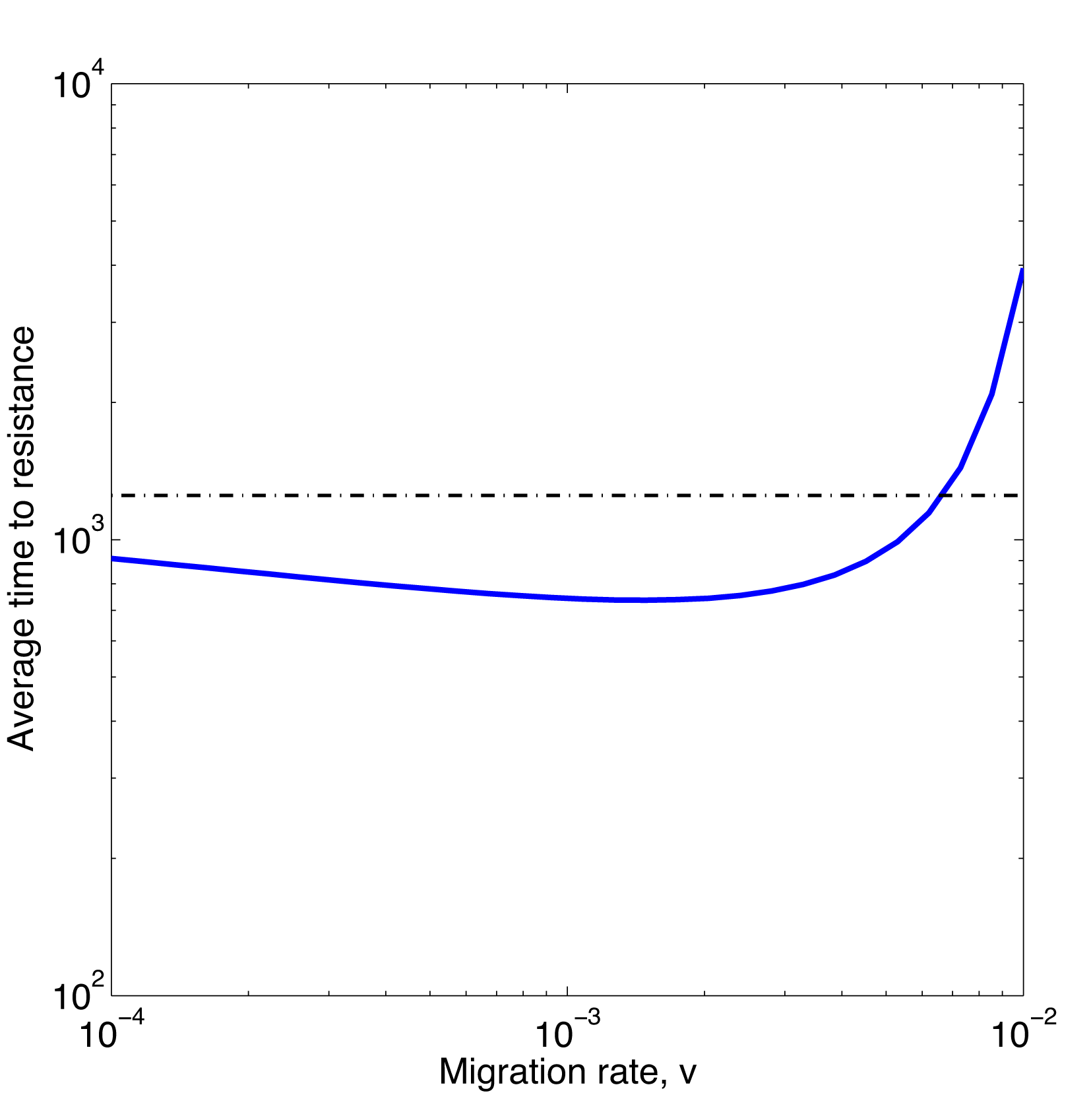

Supplement: S3 Fig — (A) shows the escape probability as a function of the difference in drug concentrations between the two metastatic compartments, ΔD. (B) shows how the average time to resistance changes with increasing ΔD. The vertical line marks the critical value of ΔD above which two point mutations are required to confer sufficient levels of resistance to increasingly high concentrations in compartment 1 while compartment 0 is the sanctuary containing lower level of drugs. Parameters: ρ = 3.5, v = 10−4, and other parameters are the same as in Fig. 4. (TIF) [file pcbi.1004142.s003.tif]

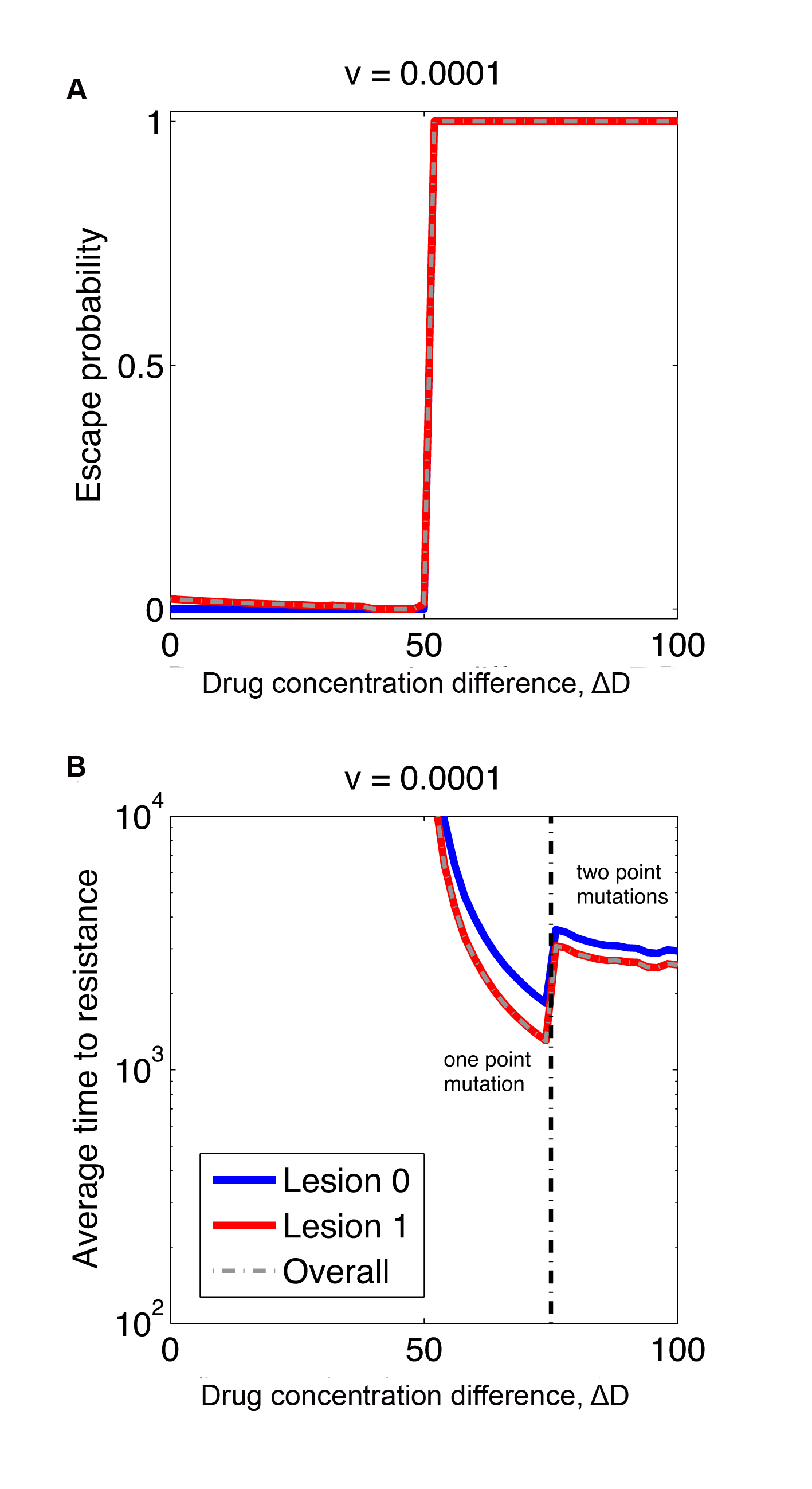

Supplement: S4 Fig — Parameters: D 0 = 0, D 1 = 100, and other parameters are the same as in Fig. 4. (TIF) [file pcbi.1004142.s004.tif]

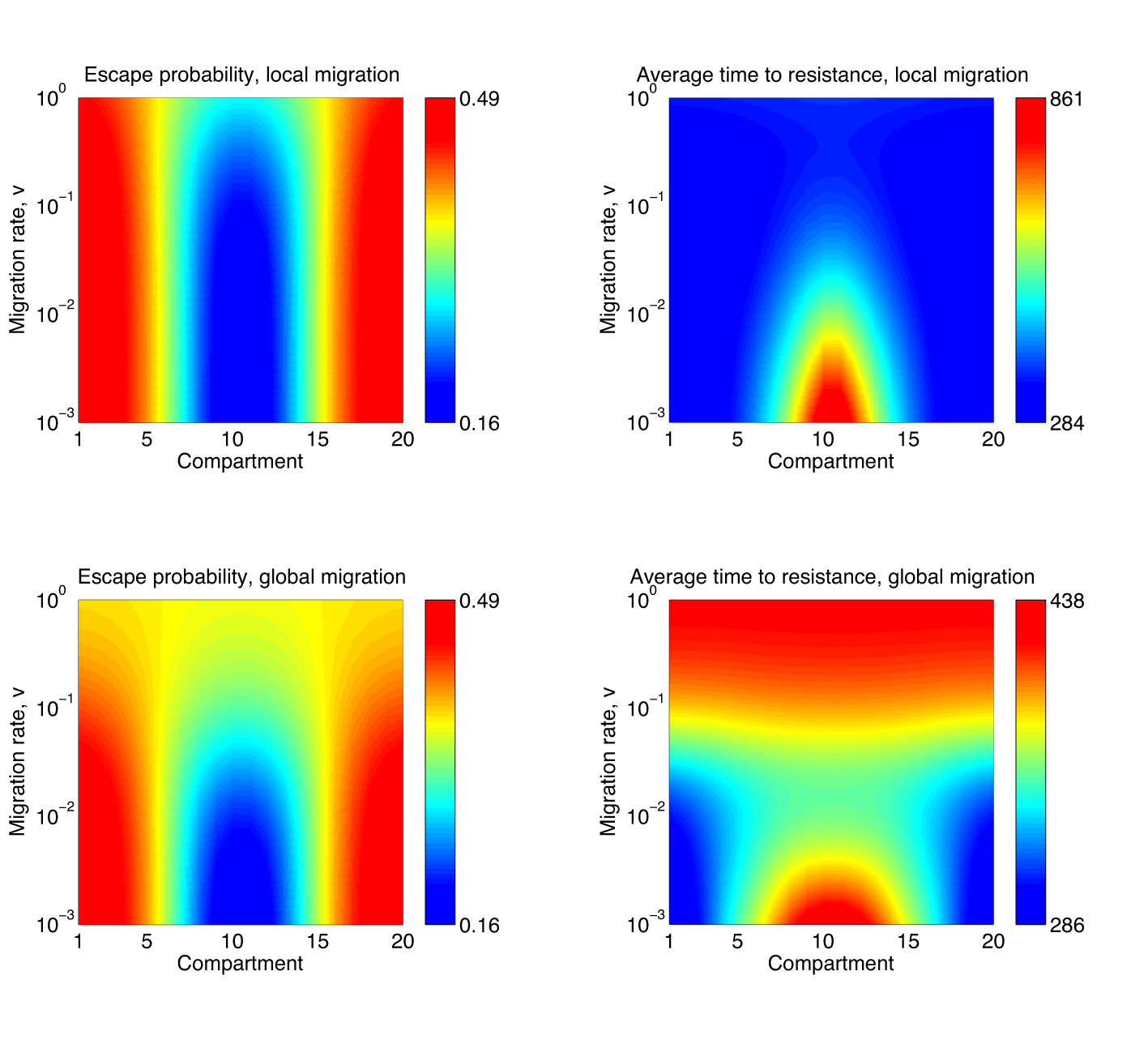

Supplement: S5 Fig — Shown are the escape probabilities and the average conditional time to resistance for a single sensitive cell initially placed in each compartment, with increasing migration rate, v. The spatial heterogeneity in drug concentrations is realized by using a rescaled Normal distribution with a peak in the central compartment, and the level of heterogeneity is denoted by the standard deviation, σ, of the concentration distribution over compartments. Parameters: M = 20, n = 5, IC50 = 100, m = 2, ρ = 1.1, D̄ = 50, s = 0.01, b 0 = 0.2, d 0 = 0.1, μ = 10−4, σ = 24.7. (TIF) [file pcbi.1004142.s005.tif]

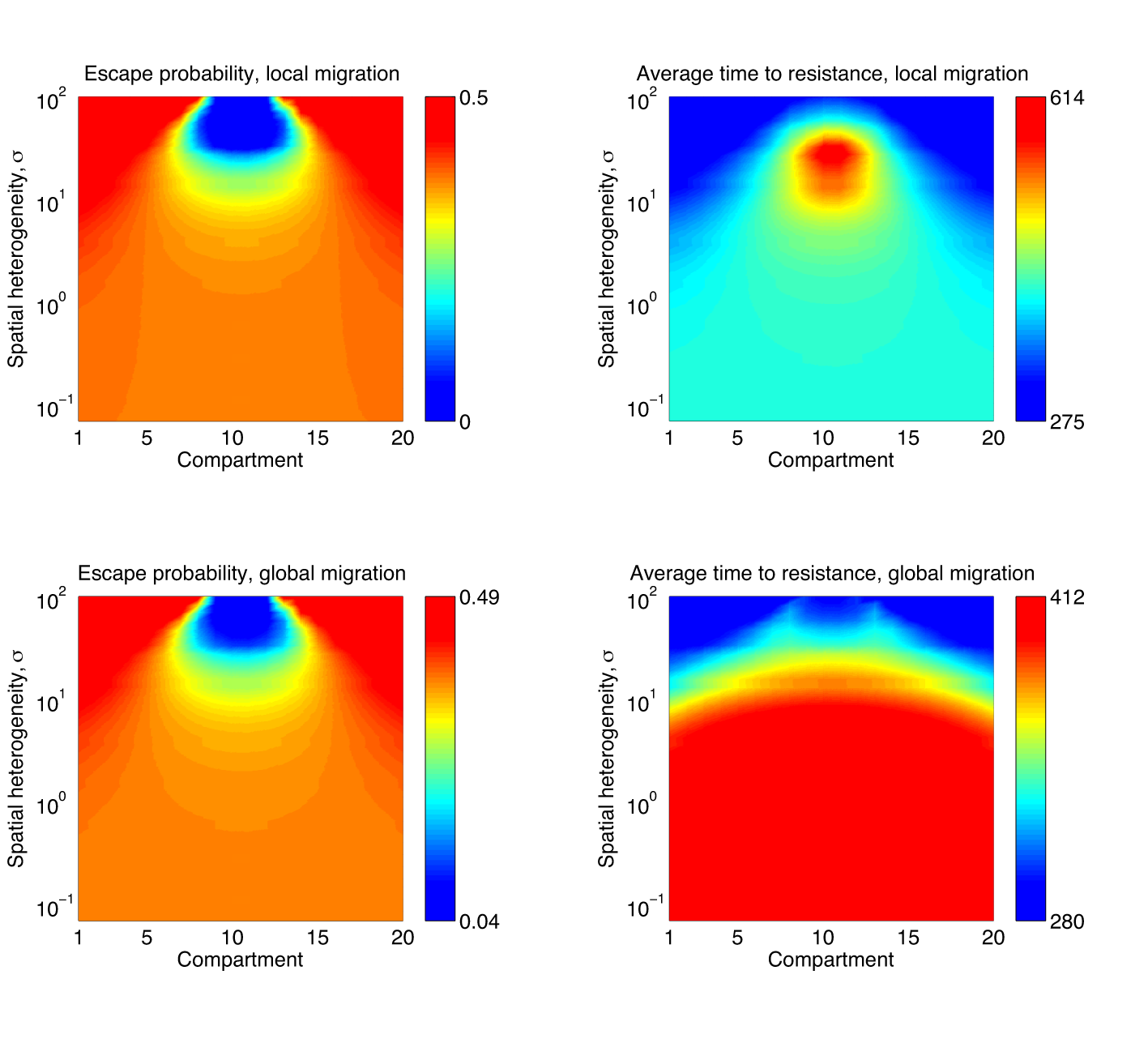

Supplement: S6 Fig — Shown are the escape probabilities and the average conditional time to resistance for a single sensitive cell initially placed in each compartment, with increasing spatial heterogeneity, σ. The spatial heterogeneity in drug concentrations is realized by using a rescaled Normal distribution with a peak in the central compartment, and the level of heterogeneity is denoted by the standard deviation, σ, of the concentration distribution over compartments. Parameters: M = 20, n = 5, IC50 = 100, m = 2, ρ = 1.1, D̄ = 50, s = 0.01, b 0 = 0.2, d 0 = 0.1, μ = 10−4, v = 0.01. (TIF) [file pcbi.1004142.s006.tif]
