# Supplementary material for: Spatial Heterogeneity in Drug Concentrations Can Facilitate the Emergence of Resistance to Cancer Therapy
Source: PLoS Comput Biol. 2015 Mar 19;11(3):e1004142. doi: 10.1371/journal.pcbi.1004142 (PMC4366398; doi:10.1371/journal.pcbi.1004142)
Supplement: S1 Text — (PDF) [file pcbi.1004142.s007.pdf]

Supplementary Information for "Spatial heterogeneity in drug concentrations can facilitate the emergence of resistance to cancer therapy"

Feng Fu, Martin A. Nowak, & Sebastian Bonhoeffer

February 5, 2015

Full model

For completeness, here we present in details the model description and the analytical methods. In this paper, we use a simple spatial model to study the evolution of resistance to cancer therapy, particularly in the presence of sanctuary sites with low or no drug concentration. Our approach is based on branching process theory. The present study focuses only on (single-) drug resistance arising during treatment, rather than pre-existing resistance prior to treatment. When cancer patients with metastatic diseases are administered potent drugs, it is often the case that the drug is not uniformly distributed throughout the body compartments of the cancer patient due to various factors such as drug penetration problems. This leads to non-homogeneous drug concentrations across different body compartments, which may harbor small metastatic populations. These compartments with low drug concentrations, which are insufficient to fully inhibit the replication of cancer cells, become the sanctuaries from which drug resistant mutants can emerge. Through migration these resistant mutants can subsequently populate compartments with high drug concentration, where the sensitive type cannot survive. By such intuitive reasoning, we hypothesize that the spatial heterogeneity in drug concentrations can speed up the evolution of acquired resistance to cancer therapy. In the rest of this supplementary information, we shall derive mathematical results to investigate this hypothesis. Our theoretical results shed new light onto the emergence of drug resistance and treatment failure of cancer patients with metastatic diseases.

Let us consider a spatial compartment model of metastatic diseases. There are M compartments in total and the drug concentration in each compartment i is denoted by D_i. We restrict

our analysis on spatially heterogeneous drug concentrations, but without time-changing concentration fluctuations. Metastatic cancer cells have already acquired motility and are assumed to migrate between compartments with rate v. The effect of the drug can either be cytostatic inhibiting cell growth and division or be cytotoxic killing cells directly, or both. Without loss of generality, we assume here that the drug inhibits cell growth. Reproduction is subject to mutation. Upon division, one of the two daughter cells mutates with rate u. By acquiring step-wise point mutations, the mutant becomes more and more adapted to drug environments and can eventually survive in high-concentration compartments. Denote by i the genotype of a cancer cell if it has acquired i point mutations. The replication rate of a cancer cell, b_{ij}, is determined by its genotype i and spatial location j as follows,

$$b_{ij} = \beta_j \frac{1 - is}{1 + \left[\frac{D_j}{\rho^i \mathrm{IC}_{50}}\right]^m}. \tag{1}$$

Here we use a Hill function for the drug response curve. β_j is the division rate of a wild type cell in compartment j in the absence of drug, s is the cost of mutation in the absence of drug, IC_{50} is the drug concentration that is needed to inhibit cell growth by one half of its original rate, ρ is the fold increase in IC_{50} per mutation, and m determines the steepness of the Hill function. We note that drug mutations are deleterious in the absence of drugs. Thus the replication rate of a mutant with genotype i and in compartment j is $(1 - is)\beta_j$ in the absence of drugs, whereas its IC_{50} is increased to $\rho^i \mathrm{IC}_{50}$ in the presence of drugs. Throughout this supplementary information, unless stated otherwise, the death rate of a cancer cell with genotype i within compartment j, d_{ij}, is all the same across compartment and equal to that of the wild type in the absence of drugs, $d_{ij} = \alpha_j = d_0$.

The migration pathway is specified by the underlying connections between compartments. For simplicity, we consider two different schemes of migration: local migration versus global migration. Local migration means that compartments are situated on a "ring" where a cancer cell can only migrate to the two nearest neighbor compartments with equal probability $v/2$. In contrast, global migration means compartments are fully connected where a cancer cell is allowed to migrate from one compartment to any other one with equal probability $v/(M - 1)$. We restrict our subsequent analysis to these two foregoing migration patterns, though it is straightforward to extend it to more complicated migration patterns in our mathematical model.

In what follows, we shall use a continuous-time multi-type branching process to describe resistance evolution.

Analysis

As for multi-type branching process, we first need to construct the appropriate type space using the combination of genotype $g_i \in \{0, 1, \cdots, n-1\}$ and compartment location $l_j \in \{0, 1, \cdots, M-1\}$. Thus a cell's type can be denoted by two-bit strings $g_i l_j \in \{0, 1, \cdots, n-1\} \times \{0, 1, \cdots, M-1\}$ and thus there are $n \times M$ different types. For example, a cell with "11"-type means that this

cell has accumulated 1 point mutation and is located in compartment 1. Therefore, a cell's type, notated by its genotype and spatial location, can change as results of genetic mutation or migration.

Next let us introduce the probability generating functions. Denote by $F_{ij}(\mathbf{X};t)$ the probability generating function for the lineages at time t initiated by a single ij-type cell, where $\mathbf{X} = [x_{00}, \ldots, x_{n-1,M-1}]$ denotes the vector of dummy variables with elements x_{ij} representing each ij-type of cells. During an infinitesimal time interval Δt, with probability $b_{ij}(1-u)\Delta t$ an ij-type cell gives birth to two identical daughter cells, with probability $b_{ij}u\Delta t$ it gives rise to an identical offspring and a mutated offspring, with probability $v\Delta t$ it migrates to another compartment, with probability $d_{ij}\Delta t$ it dies, and with probability $1-(b_{ij}+d_{ij}+v)\Delta t$ nothing changes. We can use the probability generating function approach to account for what can happen to an ij-type cell during an infinitesimal time interval Δt,

$$f_{ij}(\mathbf{X};\Delta t) = d_{ij} + b_{ij}(1-u)\Delta t x_{ij}^2 + b_{ij}u\Delta t x_{ij}x_{(i+1)j} + \frac{v}{M-1}\Delta t \sum_{k\neq j} x_{ik} + [1-(b_{ij}+d_{ij}+v)\Delta t]x_{ij}. \quad (2)$$

Using the multiplicative property of branching process (the independency of each individual lineage), the generating function $F_{ij}(\mathbf{X};t+\Delta t)$ satisfies the recursive backward equation,

$$F_{ij}(\mathbf{X};t+\Delta t) = f_{ij}(\mathbf{F};\Delta t), \quad (3)$$

where $\mathbf{F}$ is the vector of the probability generating functions with elements $F_{ij}(\mathbf{X};t)$. We obtain the following backward equation for global migration ($0 \leq i < n-1, 0 \leq j \leq M-1$).

$$\begin{aligned} \frac{\partial F_{ij}(\mathbf{X};t)}{\partial t} &= \lim_{\Delta t\to 0} \frac{f_{ij}(\mathbf{F}(t);\Delta t) - F_{ij}(\mathbf{X};t)}{\Delta t} \\ &= d_{ij} + b_{ij}(1-u)F_{ij}^2 + b_{ij}uF_{ij}F_{(i+1)j} + \frac{v}{M-1}\sum_{k\neq j} F_{ik} - (b_{ij}+d_{ij}+v)F_{ij}. \quad (4) \end{aligned}$$

Since we do not consider more than $i = n-1$ point mutations, for $i = n-1$ we have

$$\frac{\partial F_{ij}(\mathbf{X};t)}{\partial t} = d_{ij} + b_{ij}F_{ij}^2 + \frac{v}{M-1}\sum_{k\neq j} F_{ik} - (b_{ij}+d_{ij}+v)F_{ij}. \quad (5)$$

The initial condition for these ODE's is $F_{ij}(\mathbf{X};0) = x_{ij}$ for $i = 0,\ldots,n-1$ and $j = 0,\ldots,M-1$. For any given profile of the initial numbers of cells, $\mathbf{N} = \{N_{ij}\}$, the corresponding probability generating function $F_{\mathbf{N}}$ is a power compound of F_{ij}

$$F_{\mathbf{N}} = \prod_{i,j} F_{ij}(\mathbf{X};t)^{N_{ij}}. \quad (6)$$

Analogously with Eq. (4), we can derive the backward equation for local migration,

$$\frac{\partial F_{ij}(\mathbf{X};t)}{\partial t} = d_{ij} + b_{ij}(1-u)F_{ij}^2 + b_{ij}uF_{ij}F_{(i+1)j} + \frac{v}{2}F_{i[(j-1+M)\%M]} + \frac{v}{2}F_{i[(j+1)\%M]} - (b_{ij}+d_{ij}+v)F_{ij}. \quad (7)$$

In the above equation, the subscripts $[(j-1+M)\%M]$ and $[(j+1)\%M]$ account for the two neighbor compartments the cell may migrate to.

In the following unless stated otherwise, we have $d_{ij} = d_0$ for all types of cells, while b_{ij} depends on the drug concentration in compartment j as in Eq. (1). The fate of a ij-type cell can either go extinct in a relatively short time or successfully establish surviving lineages with expanding populations. The extinction probability, which is the opposite of escape probability, can be obtained by solving the fixed points of the ODE's given above. We can also calculate the probability that there is no evolved fully resistant strain in any compartment by time t, conditional on non-extinction.

For arbitrary number of genotypes and arbitrary number of compartments, we cannot find closed form probability generating functions for these nonlinear ODE's. To circumvent this difficulty, we rely on numerical ODE solvers to obtain the quantities of interest. For instance, if we solve the above nonlinear ODE's with the initial condition $x_{ij} = 0$, we can obtain the extinction probability $F_{ij}(\mathbf{0};t)$ for each ij-type cell at time t. For $t \to \infty$, $F_{ij}(\mathbf{0};t)$ converges to the steady equilibrium value (i.e., the smallest of all admissible fixed points). Using the initial condition $\mathbf{X}_{noR} = [x_{ij}]$ where $x_{ij} = 1$ for $i < n-1$ and $x_{ij} = 0$ for $i = n-1$, the conditional probability $P_{noR}(t)$ that there is no evolved fully resistant strain in any compartment by time t, starting with an ij-type cell, can be calculated as,

$$P_{noR}(t) = \frac{F_{ij}(\mathbf{X}_{noR};t) - F_{ij}(\mathbf{0};\infty)}{1 - F_{ij}(\mathbf{0};\infty)}. \tag{8}$$

In general, for any given profile of the initial numbers of cells, $\mathbf{N} = \{N_{ij}\}$, the extinction probability $P_E(t)$ at time t is given by

$$P_E(t) = \prod_{i,j} F_{ij}(\mathbf{0};t)^{N_{ij}}. \tag{9}$$

The conditional probability $P_{noR}(t)$ is given by

$$P_{noR}(t) = \frac{\prod_{i,j} F_{ij}(\mathbf{X}_{noR};t)^{N_{ij}} - \prod_{i,j} F_{ij}(\mathbf{0};\infty)^{N_{ij}}}{1 - \prod_{i,j} F_{ij}(\mathbf{0};\infty)^{N_{ij}}}. \tag{10}$$

Simple model

For the simple model with 2 genotypes and 2 compartments, we are able to derive closed-form approximations for special cases. The backward equations are as follows,

$$\begin{aligned}
\frac{\partial F_{00}}{\partial t} &= d_{00} + b_{00}(1-u)F_{00}^2 + b_{00}uF_{00}F_{10} + vF_{01} - (d_{00}+b_{00}+v)F_{00} \\
\frac{\partial F_{01}}{\partial t} &= d_{01} + b_{01}(1-u)F_{01}^2 + b_{01}uF_{01}F_{11} + vF_{00} - (d_{01}+b_{01}+v)F_{01} \\
\frac{\partial F_{10}}{\partial t} &= d_{10} + b_{10}F_{10}^2 + vF_{11} - (d_{10}+b_{10}+v)F_{10} \\
\frac{\partial F_{11}}{\partial t} &= d_{11} + b_{11}F_{11}^2 + vF_{10} - (d_{11}+b_{11}+v)F_{11}.
\end{aligned} \tag{11}$$

These are coupled nonlinear Riccati equations, for which we do not have closed-form explicit solutions.

For simplicity, let us further assume dichotomous drug environments: compartment 0 is drug free, while compartment 1 is distributed with a drug that inhibits cell growth. We have $d_{ij} = d_0$. In the drug-free compartment 0, both sensitive and resistant cells have supercritical replication potential in drug free compartment, but resistant cells have slightly lower replication rates than sensitive type due to the cost of the resistant mutation. We have $b_{00} > d_{00}$, $b_{10} > d_{10}$, and $b_{10} < b_{00}$. In the drug-containing compartment 1, sensitive cells have a subcritical replication potential while resistant cells still have a supercritical replication potential. We have $b_{01} < d_{01}$, $b_{11} > d_{11}$, and $b_{11} > b_{01}$.

For such a fitness landscape, the drug-free compartment provides far more favorable condition for breeding resistance than the drug-containing compartment. Therefore, the likely origin for treatment failure, these resistant cells that populate the drug-present compartment, should be attributed to the "mutation-migration" pathway than the "migration-mutation" pathway. In other words, it is less likely that resistance evolves *in situ* in the drug-containing compartment than that resistance mutants are originated from the drug-free compartment and then migrate to the drug-containing compartment.

Drug-environment-dependent escape

To verify these arguments above, let us first calculate and compare the likelihood of obtaining an escape mutation within one separate compartment (without any migration at all). For simplicity, the compartment is only seeded with a single sensitive cell. Denote by b_0 and b_1 the division rate of sensitive cells and resistant ones in that compartment. The death rate of the two types of cells is the same, $d_0 = d_1$. We have $b_0 > b_1 > d_0$ for compartments with low concentrations, whereas $b_0 < d_0 < b_1$ for compartments with high concentrations. Let $f^0(x, y, t)$ denote the probability generating function for the lineages starting with a single sensitive cell, and let $f^1(x, y, t)$ denote the probability generating function for the lineages starting with a single resistant cell. We have the following differential equations:

$$\frac{df^0}{dt} = d_0 + b_0(1-u)f^0 f^0 + b_0 u f^0 f^1 - (b_0 + d_0)f^0, \tag{12}$$

$$\frac{df^1}{dt} = d_1 + b_1 f^1 f^1 - (b_1 + d_1)f^1. \tag{13}$$

The initial condition is $f^0(x, y, 0) = x$ and $f^1(x, y, 0) = y$.

$b_0 > b_1 > d_0$

For sufficiently small mutation rates $u \ll 1$, we can derive a closed form approximation to the equations above. Let $r_i = b_i - d_i$ denote the net growth rate of type-i cells, $i = 0, 1$. We have

$r_0 > r_1 > 0$. Neglecting the loss of sensitive cells due to rare mutations, we have

$$\frac{df^0(x,t)}{dt} = d_0 + b_0 f^0 f^0 - (b_0 + d_0) f^0. \tag{14}$$

This ODE above is explicitly soluble, and we obtain:

$$f^0(x,t) = \frac{b_0 x - d_0 - d_0(x-1)e^{r_0 t}}{b_0 x - d_0 - b_0(x-1)e^{r_0 t}}. \tag{15}$$

Denote by $X_0(t)$ the number of sensitive cells at time t. Previous results show that $X_0(t)e^{-r_0 t}$ is a non-negative martingale and $X_0(t)e^{-r_0 t} \to W_0$ for $t \to \infty$. The Laplace transform of W_0 is given by

$$\mathbb{E}e^{-\theta W_0} = \mathbb{E}\left(e^{-\theta e^{-r_0 t}}\right)^{X_0} = f^0(e^{-\theta e^{-r_0 t}}, t). \tag{16}$$

For $t \to \infty$, using $e^{-\theta e^{-r_0 t}} - 1 \approx -\theta e^{-r_0 t}$, we obtain

$$\mathbb{E}e^{-\theta W_0} \approx \frac{r_0 + d_0\theta}{r_0 + b_0\theta} = \frac{d_0}{b_0} + (1 - \frac{d_0}{b_0})\frac{1}{1 + \frac{b_0}{r_0}\theta}. \tag{17}$$

Accordingly, we obtain the limiting distribution of W_0 for $t \to \infty$ as follows.

$$W_0 = \frac{d_0}{b_0}\delta_0 + (1 - \frac{d_0}{b_0})\text{Exponential}(\frac{r_0}{b_0}), \tag{18}$$

where δ_0 is a point mass at zero (corresponding to extinction), and Exponential($\frac{r_0}{b_0}$) is an exponential distribution with the exponent $\frac{r_0}{b_0}$. It is easy to see that, conditional on non-extinction, $X_0(t)e^{-r_0 t} \to V_0 = \text{Exponential}(\frac{r_0}{b_0})$. Thus the Laplace transform of V_0 is

$$\mathbb{E}e^{-\theta V_0} = \frac{1}{1 + \frac{b_0}{r_0}\theta}. \tag{19}$$

The rate at which resistant mutants arise is $ub_0 X_0(t)$. Hence the conditional probability that the arrival time of the first mutant $\tau_1 > t$ is

$$\begin{aligned} \mathbb{P}(\tau_1 > t | X_0(t) > 0) &= \mathbb{E}e^{-\int_0^t u b_0 V_0 e^{r_0 s} ds} \\ &= \frac{1}{1 + \frac{ub_0^2}{r_0^2}(e^{r_0 t} - 1)} \approx \frac{1}{1 + \frac{ub_0^2}{r_0^2} e^{r_0 t}}. \end{aligned} \tag{20}$$

The median arrival time

$$t_{\frac{1}{2}} = \frac{\log(r_0^2 / ub_0^2)}{r_0}.$$

We can see that $t_{\frac{1}{2}}$ is monotonically decreasing with increasing b_0 ($u \ll 1$). That is, the less harsh the compartment is ($b_0 \uparrow$), the sooner resistant mutants arise ($t_{\frac{1}{2}} \downarrow$). In this sense, compartments with lower drug concentrations are more likely the breeding ground for resistance,

since the replication of sensitive cells is less affected than in compartments with higher drug concentrations.

For small mutation rate $u \ll 1$, the escape probability $\mathbb{P}_1$ that a single sensitive cell establishes lineages without going extinction is approximately

$$\mathbb{P}_1 \approx \frac{r_0}{b_0} - u\frac{d_0(b_0 d_1 - b_1 d_0)}{b_0 b_1 r_0}. \tag{21}$$

$b_0 < d_0 < b_1$

In this unfavorable case, sensitive cells do not have a chance to survive but resistant cells do. The approximation method used above does not work here, since the branching process of sensitive cells is subcritical. To establish surviving lineages, sensitive cells must evolve resistant mutation right before quickly dying out. We cannot find a closed form for the arrival time of the first mutant for this case, but it is easy to see that if the mutant does arise (though with a lower chance), it takes much longer time due to the smaller birth rate than in benign environments. The escape probability of a single sensitive cell is approximately

$$\mathbb{P}_1 \approx -u\frac{b_0 r_1}{b_1 r_0}, \tag{22}$$

which is much smaller than Eq.(21). Moreover, we should note that the escape probability $\mathbb{P}_1$ is of the order $\sqrt{u}$ if sensitive cells are almost critical $|r_0| \to 0$:

$$\mathbb{P}_1 \approx \sqrt{u\frac{r_1}{b_1}}. \tag{23}$$

Competing pathways to the emergence of resistance

Returning to the simple model introduced above, we are now primarily concerned with which pathway leads faster to resistant cells that populate the drug-present compartment. To this end, we compare two distinct pathways. The migration-mutation pathway: sensitive cells first migrate from the drug-free compartment to the drug-present compartment, and then acquire resistance *in situ*. The mutation-migration pathway: sensitive cells acquire resistant mutation in the drug-free compartment, followed by migration to the drug-present compartment.

As shown above, we cannot obtain closed-from solutions for this four-type branching process. To simplify our mathematical derivations, let us consider unidirectional emigration flow only from the drug-free compartment to the drug-present compartment. To compare which pathway is faster, let us further artificially separate these two pathways, making only one pathway at work at a time, instead of considering them simultaneously. In this way, each pathway can seen as a three-type branching process:

$$\text{type}\,0 \xrightarrow{\mu_0} \text{type}\,1 \xrightarrow{\mu_1} \text{type}\,2.$$

For the migration-mutation pathway, $\mu_0 = v$ and $\mu_1 = ub_{01}$; for the mutation-migration pathway, $\mu_0 = ub_{00}$ and $\mu_1 = v$. The difference of the fitness landscape between these two pathways lies in the intermediate type 1, namely, the division rate b_{01} of sensitive cells in the drug-present compartment versus the division rate b_{10} of resistant cells in the drug-free compartment. It is therefore useful to first study a general three-type branching process and then obtain the results for each pathway by substituting the specific fitness landscape for each pathway.

For rare mutations ($u \ll 1$) and low migration rates ($v \ll 1$), we are able to derive explicit closed-form approximations for the conditional probability of no type 2 cells by time t. Let b_i and d_i be the birth and death rate of type i cells, $i = 0, 1, 2$. Let $r_i = b_i - d_i$ be the net growth rate of type i cells. We have $r_0 > 0$, $r_2 > 0$, and $r_1 < r_0$. Conditional on non-extinction, the number of type 0 cells at time t is given by

$$X_0(t) = V_0 e^{r_0 t} \tag{24}$$

Because type 1 is less fit than type 0 ($r_1 < r_0$), we can approximate the number of type 1 cells at time t as

$$X_1(t) = \mu_0 \int_0^t V_0 e^{r_0 s} e^{r_1(t-s)} ds = \mu_0 V_0 \frac{e^{r_0 t}[1 - e^{(r_1 - r_0)t}]}{r_0 - r_1} \sim \mu_0 V_0 \frac{e^{r_0 t}}{r_0 - r_1}, \tag{25}$$

where $x(t) \sim y(t)$ means $x(t)/y(t) \to 1$ for large t.

The conditional probability that there are no surviving type 2 cells by time t is

$$\begin{aligned}
\mathbb{P}(X_2(t) = 0 | X_0(t) > 0) &= \mathbb{E} \exp[-\mu_1 \int_0^t X_1(s) \frac{r_2}{b_2 - d_2 e^{-r_2(t-s)}} ds] \\
&= \mathbb{E} \exp[-\mu_0 \mu_1 V_0 \int_0^t \frac{e^{r_0 s}}{r_0 - r_1} \frac{r_2}{b_2 - d_2 e^{-r_2(t-s)}} ds] \\
&= \frac{1}{1 + \theta_2(t) \frac{b_0}{r_0}},
\end{aligned} \tag{26}$$

where the integral $\theta_2(t) = \mu_0 \mu_1 \frac{r_2}{r_0 - r_1} \int_0^t \frac{e^{r_0 s}}{b_2 - d_2 e^{-r_2(t-s)}} ds$.

Substituting the fitness landscape of each pathway, we find a simple condition for the mutation-migration pathway faster than the migration-mutation pathway in leading to surviving resistant cells in the drug-containing compartment if the follow inequality holds:

$$\frac{b_{00}}{b_{00} - d_{00} - (b_{10} - d_{10})} > \frac{b_{01}}{b_{00} - d_{00} - (b_{01} - d_{01})}. \tag{27}$$

Concerning cytostatic drugs that inhibits cell division, we have $d_{00} = d_{10} = d_{01} = d_{11} = d_0$, $b_{00} = b_0$, $b_{10} = (1 - s)b_0$ and $b_{01} = (1 - \delta)b_0$. s denotes the fitness cost of resistant mutation in the absence of drugs, and δ denotes the fitness cost of sensitive cells in the presence of drugs. Then the above condition simplifies to

$$\delta > \frac{s}{1 + s}. \tag{28}$$

For cells in compartments with potent drugs and with sufficiently high drug concentrations, we have $\delta > s$. More generally, the emergence of resistance is more contingent on the mutation-migration pathway than the migration-mutation pathway, if the difference of drug concentration between two neighboring compartments leads to the landscape satisfying the inequality (27).

Numerical results

In what follows, we report the numerical results on the full model. To avoid boundary problems, we consider generic, symmetric spatial distributions of drug concentrations over compartments, according to a rescaled Normal distribution such that the degree of heterogeneity can be tuned by the variance of the distribution while keeping the total sum of concentrations constant. We compare the extinction probabilities and the time to resistance for sensitive cells initially located in each compartment, with varying migration rate and the spatial heterogeneity in drug concentrations.

The results are consistent with those reported in the main text (Figs. S5 and S6). Sanctuary sites are compartments with low drug concentration, which are responsible for producing the resistance that populates compartments with high drug concentration. Selection for resistance is more contingent on the mutation-migration pathway than the migration-mutation pathway, particularly in the presence of sanctuary sites. Moreover, only for small migration rates below a certain threshold does the spatial heterogeneity in drug concentrations help speed up the resistance evolution. However, excessively high migration rates actually slow down resistance emergence, as the role of compartment structure is diminished by frequent migration.

Furthermore, we observe interesting results when comparing local migration to global migration. Both the extinction probability and the time to resistance depend more sensitively on migration rate for global migration than for local migration (Fig. S5). This is because global migration favors the dissemination of resistance all over the place, whereas local migration constrains the migration within neighboring compartments with reduced drug difference such that accruing one point mutation is sufficient to offset the increasing drug concentration. As a consequence, resistance evolves much faster for local migration than global migration, particularly for sensitive cells located at the sanctuary sites with low drug concentrations. On the contrary, the escape probability of sensitive cells located in the compartments with high concentrations is hampered more by local migration than by global migration. In this case, local migration limits its dispersal range, while global migration increases the chance of moving to sanctuary sites thus leading to greater escape probabilities. More specifically, compared to global migration, local migration speeds up the evolution of resistance for sensitive cells initially located at sanctuary sites, while delaying the evolution of resistance for sensitive cells initially located at harsh compartments with high levels of drugs. Regarding the latter case, moreover, there exists an optimal intermediate global migration rate with which the time to resistance is minimal.

Sanctuary sites for sensitive cells result when varying from homogeneous drug distributions to increasingly heterogeneous drug concentrations across spatial compartments. It is obvious

that compartments with low concentrations become the shelter for sensitive cells and thus provide benign condition for breeding resistance that can be selected for in compartments with excessively high concentrations (Fig. S6). Local migration takes less time to evolve resistance than global migration, if these cells are initially located at the sanctuaries. Opposite results are obtained if these cells are initially located at harsh compartments. Under local migration, there exists an optimal level of heterogeneity in drug concentrations that most delays the emergence of resistance. In contrast, increasing the spatial heterogeneity in drug concentrations helps prolong the time to resistance under global migration.

Altogether, these results confirm that our conclusions derived from the simple model in the main text are robust with respect to model extensions and varying model parameters. In addition, the scheme of migration plays an important role in the evolution of acquired resistance. However, future studies are needed in order to characterize, and eventually manipulate, the migration routes for metastatic cells. We think that constraining escape route of metastatic cells to sanctuary sites and limiting dissemination of evolved metastatic cells from sanctuary sites are vital to eliminate disseminated cancer.
